# Social inequalities in climate change-attributed impacts of Hurricane Harvey

**DOI:** 10.1038/s41467-022-31056-2

**Published:** 2022-08-25

**Authors:** Kevin T. Smiley, Ilan Noy, Michael F. Wehner, Dave Frame, Christopher C. Sampson, Oliver E. J. Wing

**Affiliations:** 1 Department of Sociology, Louisiana State University, Baton Rouge, LA USA; 2 School of Economics and Finance, Victoria University of Wellington, Wellington, New Zealand; 3 Computational Research Division, Lawrence Berkeley National Laboratory, Berkeley, CA USA; 4 School of Geography, Environmental and Earth Science, Victoria University of Wellington, Wellington, UK; 5 Fathom, Bristol, UK

**Keywords:** Climate-change impacts, Socioeconomic scenarios, Sociology, Attribution, Geography

## Abstract

Climate change is already increasing the severity of extreme weather events such as with rainfall during hurricanes. But little research to date investigates if, and to what extent, there are social inequalities in climate change-attributed extreme weather event impacts. Here, we use climate change attribution science paired with hydrological flood models to estimate climate change-attributed flood depths and damages during Hurricane Harvey in Harris County, Texas. Using detailed land-parcel and census tract socio-economic data, we then describe the socio-spatial characteristics associated with these climate change-induced impacts. We show that 30 to 50% of the flooded properties would not have flooded without climate change. Climate change-attributed impacts were particularly felt in Latina/x/o neighborhoods, and especially so in Latina/x/o neighborhoods that were low-income and among those located outside of FEMA’s 100-year floodplain. Our focus is thus on climate justice challenges that not only concern future climate change-induced risks, but are already affecting vulnerable populations disproportionately now.

## Abstract (web summary)

New study shows that up to 50% of properties flooded after hurricane Harvey flooded because of climate change, with low-income and Latina/x/o neighborhoods experiencing higher climate change-attributed impacts.

## Introduction

Climate change can increase the intensity of extreme weather events such as the amount of rainfall associated with tropical storms and cyclones. Climate change can therefore worsen the impact of these events and may do so in unequal ways. Indeed, research has already separately identified unequal social vulnerabilities in flood risks regardless of climate change^1–4^, and increasing flood risks from climate change^5–8^. But, not much has been done to connect these two insights. Specifically, climate change’s precise role in shaping unequal social impacts now is not yet well-understood^6^. This is our focus here, where we combine extreme weather event attribution (to climatic change) research together with spatial quantitative social research.

This type of event attribution seeks to determine how the meteorological and environmental characteristics for specific extreme weather events that have already occurred were shaped by anthropogenic changes to the climate^5,9–14^. As such, it sheds light on how climate change has affected both the likelihood and the intensity of these events. A new strand of this work now melds extreme weather event attribution with hydrological models to estimate the spatial imprint of these events’ impacts ^15,16^.

While this growing work on attribution disentangles climate change’s role in extreme weather hazards, no research to date analyzes if and to what extent these impacts of the increasing hazard link to pre-existing social inequalities. Here, we build on social science work identifying inequalities in disaster impacts that invokes oft-cited, but little-tested hypotheses about increasingly severe and frequent disasters because of climate change. To do so, we empirically assess the increased severity of disaster impacts because of climate change by focusing on the distribution of these climate change-attributed impacts across different social groups. To do so, we synthesize data on climate change attribution, hydrological flood models, hazard maps, and socio-spatial characteristics of neighborhoods and land parcels in Harris County, Texas during Hurricane Harvey in 2017.

In this work, we first examine the extent to which flooding of residential buildings from Hurricane Harvey could be attributed to climate change^16^. Then, using multivariable econometric regression models, we assess what social and demographic factors are associated with these climate change-induced impacts, thereby carrying out an original analysis of inequalities in climate change-attributed impacts of extreme weather events. Our analysis is based on a census of approximately 1.1 million residential land parcels located within 795 census tracts (i.e., neighborhoods) in Harris County, Texas—the largest county of the Houston metropolitan area that was among the hardest-hit areas by Hurricane Harvey^17–19^.

## Results

### Climate change-induced impacts of Hurricane Harvey

To determine the relative share of flood impacts during Hurricane Harvey attributable to climate change, we calculated climate change-attributed depths and damages using scenarios that compare the flooding that actually occurred to scenarios of flooding with less precipitation (i.e., flooding without climate change). Damages were calculated using depth-damage relationships specific to the building type, as defined in the National Structural Inventory (NSI) data. The mean damage was calculated for each structure in our dataset using the nonlinear damage functions (damage as a function of flood depths) constructed from National Flood Insurance claims data and the NSI types, as described in ref. ^20^.

Previous research examined seven possible scenarios of 7, 8, 13, 19, 20, 24, and 38% of precipitation during the storm that could be attributed to climate change; see ref. ^16^ and Methods for details. We calculate the climate change-attributed portion of depths and damages by subtracting flooding data from the scenarios with less precipitation from the baseline flood that occurred. Here, we present results for the two “best estimates”; a lower scenario of 20% less precipitation without climate change (the “best estimate” from ref. ^13^ and similar to the multigroup best estimate average of 19% from ref. ^9^), and a higher one of 38% less precipitation (the “best small-region estimate” from ref. ^10^). Results for the other five scenarios are presented in the Supplementary Information in Tables S1–S13. Results are shown for residential parcels.

Our analysis shows that 9.7 percent of residential parcels (~106,000 parcels) had buildings that flooded during Hurricane Harvey. For all seven climate change-attribution scenarios we consider, almost every flooded building (>99%) experienced at least some flooding attributed to climate change. These depths varied: the median increased flood depths attributed to climate change was 22 cm in the (20%) lower climate change-attribution scenario, and 27 cm in the (38%) higher scenario.

**Fig. 1:**
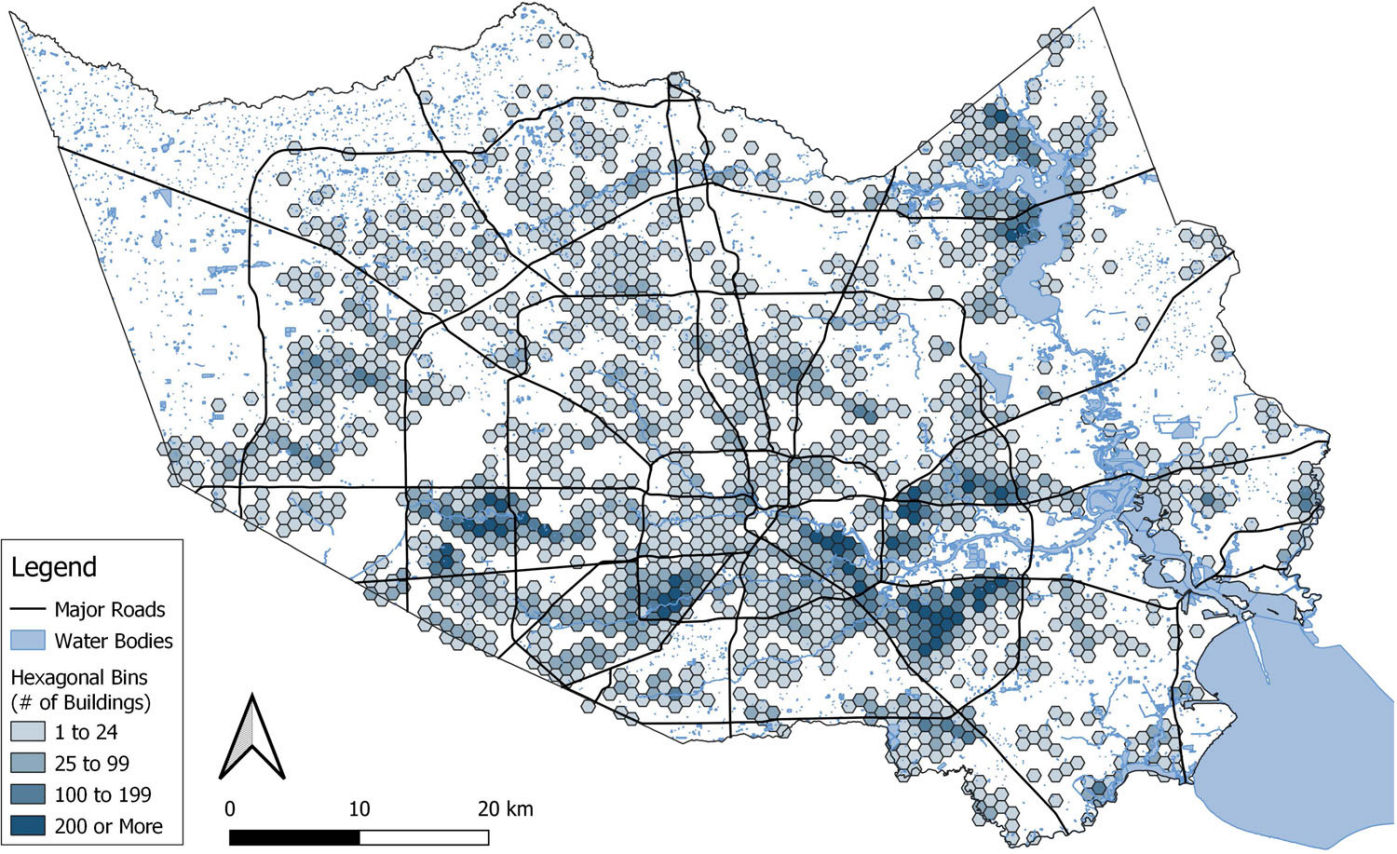
Map of climate change-attributed flooding (38% scenario). Each hexagonal bin symbolizes the number of residential buildings that would not have flooded without the added impact of climate change in Harris County, Texas during Hurricane Harvey.

These climate change-attributed flood depths often made the difference between flooding a building and not flooding the same building at all. In the higher scenario (38% of precipitation is attributable to climate change), 49.4% of the buildings that were flooded would have been flooded anyway, but 50.6% flooded only because of climate change; i.e., they would not have been flooded during the hurricane had there been no anthropogenic climate change to generate increased rainfall. Since Harris County is large, this corresponds to an estimated 53,616 parcels that would not have been flooded without climate change. Figure 1 shows a map of areas that experienced flood impacts only because of climate change in the 38% scenario. For the lower “best estimate” (20%), the comparable figure is almost a third—i.e., 31.9% of the flooded houses would not have flooded without climate change. Even in the most conservative scenario we test—only 7% of the precipitation is associated with climate change—12.8% of the flooded residential buildings would not have flooded at all without climate change.

We also calculate the property damages wrought by these climate change-attributed flood depths using the information on buildings from the National Structure Inventory and depth-damage functions outlined in ref. ^20^ (see Methods). Our modeled estimate of the baseline flood damage to residential properties, including flooding both attributed and not attributed to climate change, is US$ 6.41 billion in Harris County. We estimate the climate change-attributed portion of these damages to be approximately $2.39 billion (37.2%) of total damages in the lower scenario or $3.7 billion (57.8%) in the higher climate change scenario.

### Analysis of climate change-attributed impacts

Given the sizeable impacts of climate change on residential flooding from Hurricane Harvey, we next conduct regression analyses assessing what social and demographic characteristics of neighborhoods and land parcels are associated with these climate change-attributed impacts. We analyze neighborhood-level variables including the racial composition and median income of the census tracts, and including potentially noteworthy moderating (interacting) relationships between racial composition and income. We also examine parcel-level variables including the parcel’s appraised value, whether it is a single-family residential home, a mobile home, or a multifamily home, the year the residential structure was built, and whether the parcel has a building located in FEMA’s 100-year floodplain. In these regressions, statistically significant relationships and effect size calculations can be interpreted to identify disproportionate impacts for a social or demographic group.

Our first set of regressions assesses how these characteristics relate to two dependent variables attributed to climate change^1^: flood depths (in cm for buildings where flooding was >20 cm); and ref. ^2^ flood damages (the estimated amount of damage to residential buildings in U.S. dollars). The multivariable regressions use a Tobit specification, as a Tobit regression is appropriate for a left-censored variable where there are a large number of 0 cases (because many parcels did not have climate change-attributed flood depths). Table 1 shows these results for the 20 and 38% scenarios.

**Table 1:** Tobit regression of climate change-attributed depths for Hurricane Harvey in Harris County, Texas.

	(1)	(2)	(3)	(4)
	Depths: 20% scenario	Depths: 38% scenario	Damages: 20% scenario	Damages: 38% scenario
Mobile homes (ref. single-family homes)	−0.146^*^ (0.067)	−0.244^*^ (0.104)	−13974.716 (8660.438)	−22485.544 (12907.822)
Multifamily residences (ref. single-family homes)	−0.286^***^ (0.037)	−0.455^***^ (0.060)	−27291.861^***^ (3751.408)	−41537.089^***^ (5786.324)
Appraised value (in 10,000s)	0.000^*^ (0.000)	0.000^**^ (0.000)	78.956^***^ (13.389)	118.819^***^ (19.783)
Appraised value (in 10,000s)*Appraised value (in 10,000 s)	−0.000 (0.000)	−0.000 (0.000)	−0.008^***^ (0.002)	−0.012^***^ (0.003)
FEMA 100-year floodplain	0.300^***^ (0.027)	0.484^***^ (0.044)	39172.316^***^ (4115.806)	59673.766^***^ (6242.381)
Year built	−0.004^***^ (0.001)	−0.006^***^ (0.001)	−486.906^***^ (69.003)	−731.664^***^ (106.573)
Prop. Latina/x/o	0.838^***^ (0.192)	1.341^***^ (0.312)	96245.978^***^ (22228.551)	146089.445^***^ (33922.639)
Prop. Black	−0.492 (0.277)	−0.798 (0.446)	−55625.818 (32584.418)	−83405.492 (49818.786)
Prop. other race	−0.172 (0.515)	−0.285 (0.828)	−25886.556 (59985.583)	−41412.489 (91617.440)
Median income (in 10,000s)	0.043^*^ (0.018)	0.068^*^ (0.030)	4558.075^*^ (2006.640)	6843.985^*^ (3075.339)
Prop. Black*median income (in 10,000s)	0.061 (0.067)	0.098 (0.108)	8795.172 (7973.184)	12926.780 (12139.877)
Prop. Latina/x/o*median income (in 10,000s)	−0.113^*^ (0.049)	−0.180^*^ (0.079)	−11922.360^*^ (5478.747)	−17647.823^*^ (8414.736)
Prop. other race*median income (in 10,000s)	−0.092 (0.080)	−0.147 (0.130)	−9267.221 (8833.474)	−13570.820 (13470.497)
Constant	6.402^***^ (1.100)	10.166^***^ (1.783)	841824.157^***^ (134170.769)	1263358.957^***^ (207126.315)
Observations	1108198	1108198	1108198	1108198

Standard errors in parentheses.

Standard errors clustered within census tracts.

Statistical tests are two-sided.

^*^p < 0.05, ^**^p < 0.01, ^***^p < 0.001

**Fig. 2:**
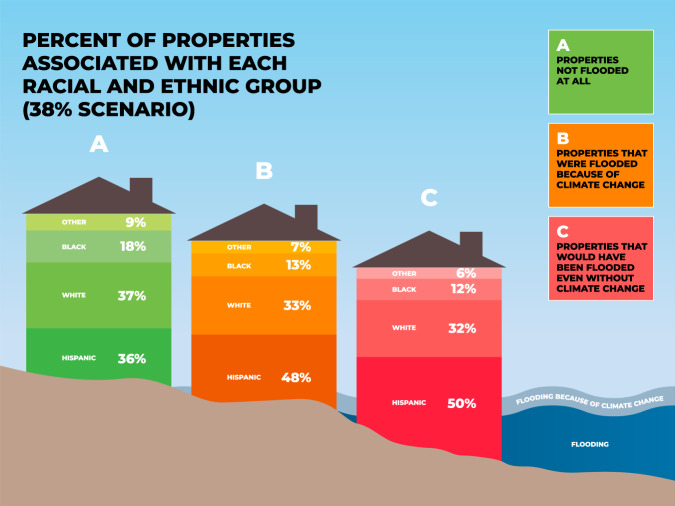
Percent of properties associated with each racial and ethnic group (38% scenario). Estimated percentages for residential properties in Harris County, Texas during Hurricane Harvey. Note: Group A included 1,002,026 parcels, group B 53,616 parcels, and group C 52,439 parcels.

**Fig. 3:**
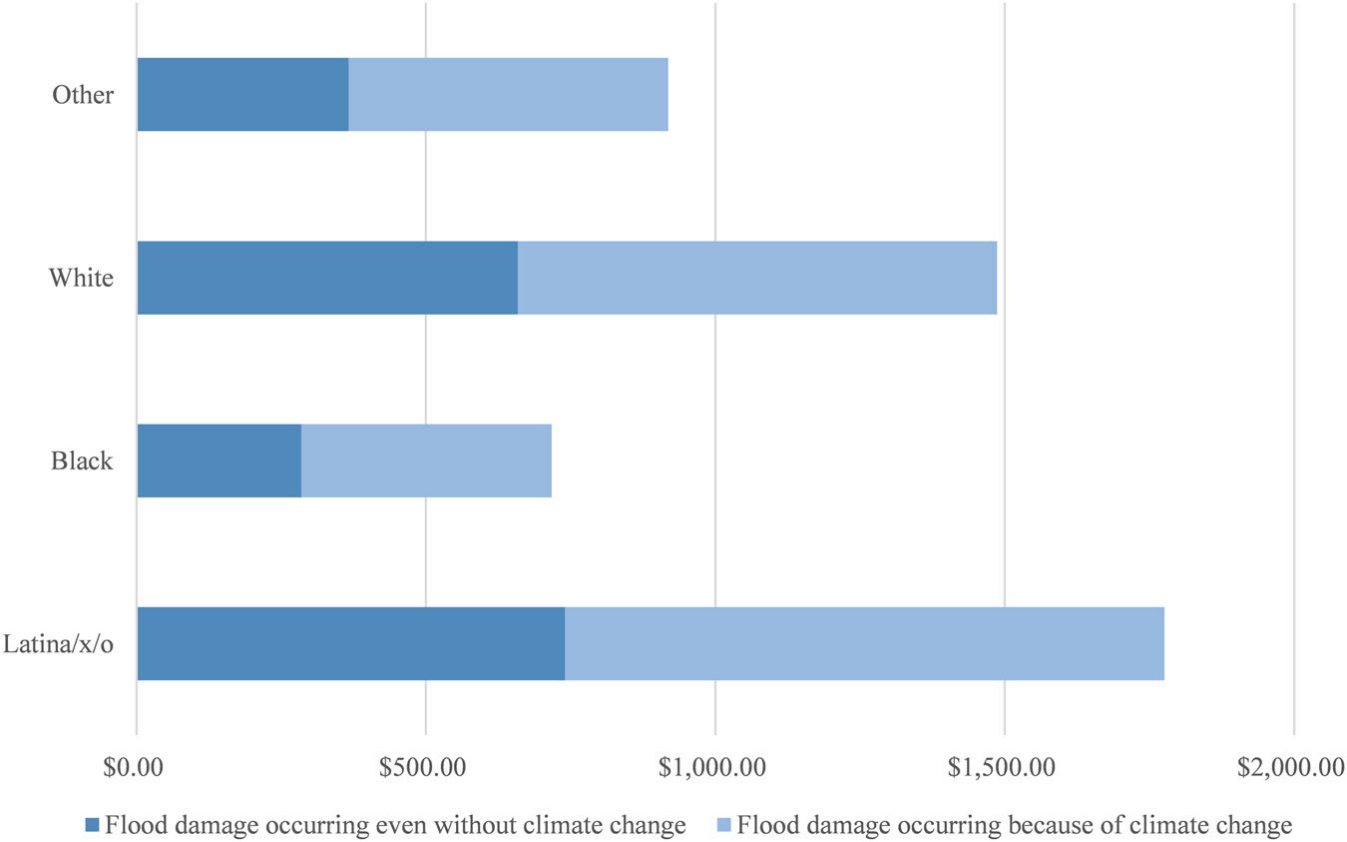
Estimated per capita property damage from flooding by racial composition (38% scenario). Estimated per capita damages for residential properties in Harris County, TX during Hurricane Harvey.

We identify six primary findings from these analyses that hold across the different scenarios for both depths and damages. First, parcels in neighborhoods with more Latina/x/o residents had higher climate change-attributed impacts. Figure 2 uses descriptive statistics where we multiply the number of parcels in three categories (i.e., not flooded, flooded because of climate change, would have flooded even without climate change) with the proportion of different racial groups in each neighborhood to provide a schematic to illustrate the racial disparities in flood depths for the 38% scenario. Figure 3 uses descriptive statistics (in the same manner as Fig. 2) to show these disparities for damages, with the per capita damages for a Latina/x/o person from climate change-attributed flooding estimated at ~$1,035. Although this estimate is only narrowly higher than that for whites ($828), it should be noted that home values are higher in white neighborhoods, and therefore this disparity per unit of home value is greater^21–23^.

Second, parcels in neighborhoods with higher incomes had higher climate change-attributed impacts. Third, in neighborhoods with more Latina/x/o residents, the impact of income is reversed. In these neighborhoods, a greater impact was observed in the lower-income neighborhoods. This finding clarifies the previous two: While greater neighborhood incomes are linked to more climate change-induced impacts, the opposite is the case in Latina/x/a neighborhoods. Fourth, multifamily residential parcels (compared to single-family parcels) experienced less flood impacts associated with climate change. Fifth, location in FEMA’s 100-year floodplain was linked to greater climate change-attributed impacts. Sixth, older residential structures tended to have greater flood impacts.

In addition to these primary findings, other results were less consistent. For climate change-attributed damages but not climate change-attributed depths, we found evidence of a curvilinear (convex) effect for the appraised value of the parcel, but effect sizes were relatively small. We also found that mobile homes experienced less flood depths but this was not statistically significant for flood damages. Finally, we did not find statistically significant relationships for census tracts with a high proportion of non-Latina/x/o blacks or non-Latina/x/o of other races, including for moderating relationships with income.

### Analysis of flooding only because of climate change impacts

In the second set of regression analyses, we ask what social and demographic characteristics are linked to parcels that would not have flooded without climate change by transforming our flood depths and flood damages variables into binary outcomes that denote whether a parcel’s buildings would not have flooded or did not flood at all. Parcels that would have flooded even without climate change-attributed precipitation are excluded, meaning that we conceptualize the sample as all parcels that would not have had flooded buildings if not for climate change, and then distinguish between those that did or did not flood in the climate change scenarios we examine. Table 2 shows the results of these binary logistic regression analyses.

**Table 2:** Logistic regression of climate change-attributed depths for Hurricane Harvey in Harris County, Texas.

	(1)	(2)	(3)	(4)
	Depths: 20% Scenario	Depths: 38% Scenario	Damages: 20% Scenario	Damages: 38% Scenario
Mobile homes (ref. single-family homes)	0.678 (0.222)	0.701 (0.252)	0.673 (0.221)	0.704 (0.254)
Multifamily residences (ref. single-family homes)	0.289^***^ (0.046)	0.283^***^ (0.041)	0.319^***^ (0.049)	0.310^***^ (0.043)
Appraised value (in 10,000s)	1.001 (0.001)	1.001 (0.001)	1.001^**^ (0.001)	1.001^**^ (0.001)
Appraised value (in 10,000s)*Appraised value (in 10,000s)	1.000 (0.000)	1.000 (0.000)	1.000 (0.000)	1.000 (0.000)
FEMA 100-year floodplain	3.267^***^ (0.384)	3.553^***^ (0.437)	3.262^***^ (0.384)	3.546^***^ (0.436)
Year built	0.984^***^ (0.002)	0.984^***^ (0.002)	0.984^***^ (0.002)	0.984^***^ (0.002)
Prop. Latina/x/o	21.542^***^ (13.672)	22.893^***^ (15.104)	20.782^***^ (13.154)	22.090^***^ (14.551)
Prop. Black	0.109 (0.126)	0.141 (0.174)	0.110 (0.126)	0.141 (0.173)
Prop. other race	0.362 (0.646)	0.610 (1.189)	0.333 (0.590)	0.563 (1.094)
Median income (in 10,000s)	1.160^**^ (0.056)	1.170^**^ (0.064)	1.153^**^ (0.056)	1.163^**^ (0.064)
Prop. Black*median income (in 10,000s)	1.569 (0.424)	1.458 (0.431)	1.569 (0.423)	1.461 (0.432)
Prop. Latina/x/o*median income (in 10,000s)	0.675^*^ (0.116)	0.712 (0.131)	0.681^*^ (0.117)	0.717 (0.132)
Prop. Other race*median income (in 10,000s)	0.811 (0.180)	0.769 (0.191)	0.822 (0.182)	0.778 (0.194)
Observations	1036024	1055759	1035536	1055313

Exponentiated coefficients; Standard errors in parentheses.

Standard errors clustered within census tracts.

Statistical tests are two-sided.

^*^p < 0.05, ^**^p < 0.01, ^***^p < 0.001

**Fig. 4:**
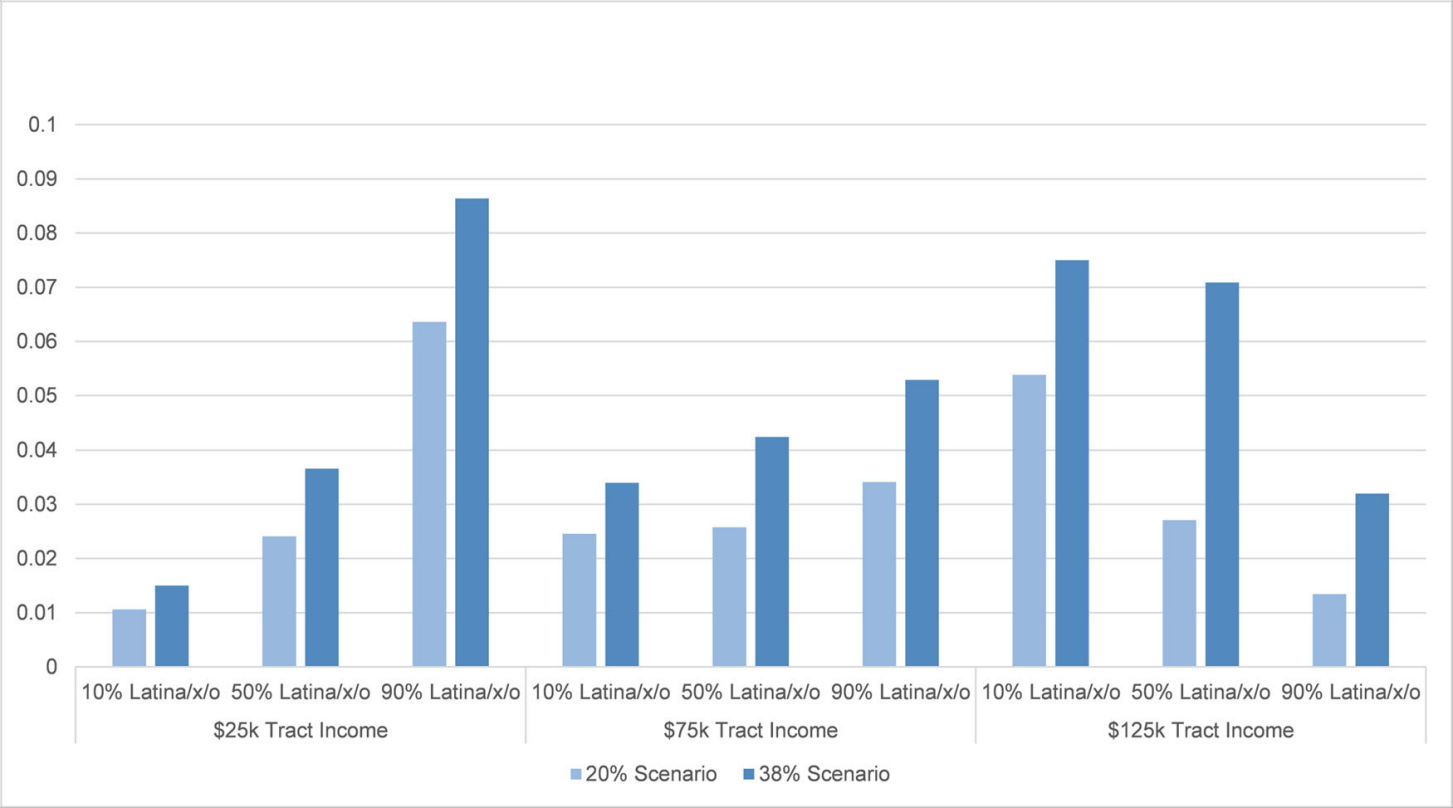
Predicted probabilities of parcel flooding only because of climate change. Predicted probabilities calculated for binary logistic regression results in Table 2 at levels of percent Latina/x/o, for Hurricane Harvey in Harris County, TX.

Findings from these logistic analyses largely mirror those from the Tobit models on climate change-attributed flood depths and damages, thereby providing robust support for the overall findings. Most central to this study’s focus on climate justice, we find that Latina/x/o neighborhoods, especially low-income Latina/x/o neighborhoods, had greater odds of flooding (compared to other types of neighborhoods) only because of the added climate change-induced precipitation. This finding held for both depths and damages across each of the climate change-attribution scenarios, although the interaction effect for Latina/x/o and median income is slightly smaller in models for the 38% scenario (where *p* values are 0.064 for Model 2 and 0.07 for Model 4). Figure 4 graphs these findings with predicted probabilities by estimating the percentage of climate change-only flooded properties at different population shares of Latina/x/o residents and median income. As an example, the estimates show that for a high Latina/x/o population share, low-income (90%, $25,000 median income) neighborhood, we would estimate that ~9% of parcels in the 38% scenario (and 6% in the 20% scenario) would not have flooded if not for climate change.

Four additional findings are also similar to the Tobit regression findings. First, a parcel’s location in a higher-income neighborhood is associated with higher odds of that parcel flooding only because of climate change. While this finding suggests greater hazard exposure for residents living in neighborhoods that are more economically well-off, it is also in juxtaposition to the opposite effect for income found in Latina/x/o neighborhoods. Second, multifamily residential parcels (compared to single-family parcels) had lower odds of crossing the flooding threshold of 20 cm because of climate change-induced impacts. Third, parcels located inside the FEMA 100-year floodplain had greater odds of climate change-attributed flooding. Fourth, older residences had higher odds of flooding only because of climate change.

### Analysis inside and outside of floodplains

In the third set of analyses, we ask how social inequalities in climate change-attributed impacts are linked to the location in FEMA-delineated 100-year floodplains. Location in this Special Hazard Flood Area (SFHA) is the primary indicator of flood risk in the United States. For instance, any property within the 100-year flood zone is required to purchase flood insurance through the National Flood Insurance Program (NFIP) in order to be eligible for a mortgage from a federal agency^24^. Properties outside the SFHA, in contrast, are not required to purchase the NFIP coverage. Nevertheless, many homeowners within the SFHA still do not have flood insurance^25–27^. These uninsured may be undertaking other strategies to mitigate damage from flooding. More broadly, at the very least these within the SFHA are made aware that their residence is significantly exposed to flood risk. By contrast, residents outside of the SFHA are not similarly warned, and may therefore perceive a lower (or even nonexistent) risk in their outside-the-SFHA locations, even if the risk they face may also be significant^27,28^.

Our descriptive analyses show large impacts outside of the 100-year floodplains: 76.1% of flooded parcels are located outside of the SFHA floodplains, an impact totaling $4.9 billion in damages. The climate change-attributed portion of damages is higher outside of the floodplains (38.5% of damages in the lower scenario and 59.5% in the higher scenario) than inside of the floodplains (33.2% of damages in the lower scenario and 52.2% in the higher scenario). Coupling these climate change-attributed impacts with SFHA floodplain location, we estimate that between 29 and 45% of all damages from Harvey (totaling $1.9 to $2.9 billion in our model) occurred because of climate change and outside of the floodplain.

**Fig. 5:**
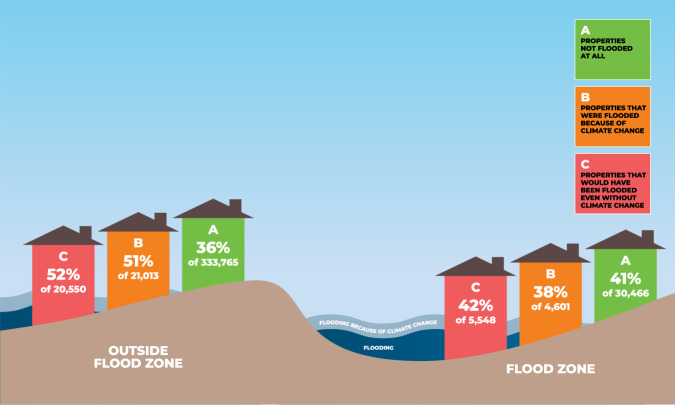
Percent of Latina/x/o parcels flooded because of climate change inside and outside of floodplains (38% scenario). Estimated percentages for residential properties in Harris County, Texas during Hurricane Harvey.

We find evidence for social inequalities in climate change-attributed impacts outside of the floodplain, but less so inside the floodplain. We re-analyzed the Tobit and binary logistic regression models in the two previous sections to account for a moderating relationship between floodplain location and census tract-level racial composition and median income variables; results are found in the Supplementary Information in Tables S9–S13. Among parcels outside of the floodplains, the econometric models show that climate change-attributed flooding is more likely in census tracts with more Latina/x/o residents. Previous findings relating to income, proportion Latina/x/o, and the moderating effect between these two variables hold in these models. In Fig. 5, we estimate (using descriptive statistics in a similar approach to that of Fig. 2) that ~52% of all parcels outside of the floodplain flooded because of climate change are estimated to be Latina/x/o households compared to 38% inside of the floodplain.

Taken together, these findings suggest that there are more pronounced inequalities in climate change-attributed impacts in flooding outside of FEMA’s 100-year SFHA floodplains. Floodplain location is a key policy tool used to attempt to compel the uptake of flood insurance and other flood mitigation measures. This is important, as the insured homeowners, or those that were forewarned, are more likely to have the resources to pay for reconstruction and recovery. As such, a house located outside the floodplain is less likely to have access to recovery funding and is less likely to recover well^29–32^. Thus, the racial inequalities we find in the damage can be further exacerbated during the disaster recovery process.

## Discussion

Drawing on flood impacts from Hurricane Harvey in Harris County, Texas, our analysis finds strong evidence for climate change-attributed flooding: we estimate that 30 to 50% of properties in Harris County would not have flooded if not for the increased rainfall due to climate change. Importantly, we also find evidence for social inequalities in this climate change-attributed flooding. Most notably, parcels in Latina/x/o neighborhoods disproportionately experienced these higher levels of flooding. Within Latina/x/o neighborhoods, parcels located in low-income neighborhoods were even more likely to experience these impacts, and, among parcels located outside of the FEMA floodplain, Latina/x/o neighborhoods were also more likely to experience impacts. All of these suggest that low-income Latina/x/o neighborhoods were more likely to be exposed to climate change-attributed flooding in Hurricane Harvey, at the same time that residents in Latina/x/o neighborhoods were also less likely to be forewarned of the flood risk to which they may be exposed, as they were less likely to be located in the SFHA floodplains. Being warned could have compelled the uptake of flood insurance or alternatively to adapt to this risk using other mitigation strategies. These findings are partly in contrast to our findings for neighborhood median income which show that more economically well-off neighborhoods experienced greater impacts. These patterns were evident across all the climate change scenarios we examined and for the various damage measures we tracked: for flood depths, for flood damages, and for whether the flooding would have occurred without climate change-attributed an increase in flood depths.

Our findings offer a window not only into the climate justice challenges that cities and towns may face in the future because of climate change, but the challenges these communities already confront right now. The world has warmed by more than 1 °C already^33^. The common frame of analyzing how climate change might have inequitable social impacts in the future is important but incomplete. We believe an equally important line of inquiry should focus on how climate change is already having unequal social impacts now. Already, this theme is emergent in research on topics like climate migration^34–36^. We believe that social science analysis of climate change-attribution impacts of extreme weather events can further substantiate the major (and inequitable) impacts climate change is having in our world today.

The specific climate justice challenges are animated by residential segregation (by race and income) in the United States and along other lines of social differentiation around the world. Our findings suggest that this socio-spatial inequality is linked to the increasing impacts of climate change. Following this, one important implication of our work is that climate change could exacerbate social inequalities in the wake of extreme weather events, if hard-hit areas are majority-minority areas, and especially if they are low-income or otherwise socially or economically vulnerable. Hurricane Harvey is exactly such a case.

In many instances, inequalities persist after the damage has been incurred, during the process of recovery^37^. The recovery of lower-income and/or minority communities is often slower and less complete or successful. Previous research has shown, for instance, that lower-income households are less likely to be insured, or receive lower compensation from their insurer, *ceteris paribus*^26,28,32^. Indeed, insurance is often essential for a fast recovery^38^. More than this, the binary nature of risk determination (i.e., location in a 100-year floodplain—or not) in U.S. flood policy would suggest that location outside of the 100-year floodplain would be linked to lower perceived risk^6,32^ and would experience a more difficult recovery. Indeed, Billings et al^39^. find that for the aftermath of Hurricane Harvey. Our finding that among land parcels outside of the floodplains Latina/x/o neighborhoods disproportionately experienced climate change-induced impacts suggests that these differentials in risk determination, risk messaging/signaling, and the plausible likelihood of having flood insurance could all further amplify inequalities during the disaster recovery process.

Parallel to this, an additional thread to examine is how neighborhood economic characteristics like median income relate to climate change-induced impacts. Higher-income areas are often closer to water bodies as these are perceived as desirable amenities^40,41^. In Harris County, our findings can be partly interpreted through this lens, as proximity to water bodies can pattern on affluence in some areas (such as along recreational trails on the city’s many bayous) but have the opposite effect in others (such as with low-income Latina/x/o neighborhoods near the Houston Ship Channel which hosts a large number of petrochemical facilities)^40–42^. Thus, increasing attention must be paid not only to both climate change mitigation and adaptation to lessen these impacts but also to upending the root social inequalities that sustain socio-spatial differentiation in the first place.

If and to what extent social inequalities in climate change-attributed impacts may hold across places and across other types of extreme weather events, however, is a critical question for future research^43^. Our study is of a single extreme weather event in a single location, and we are agnostic as to the external validity of our findings. Is our work generalizable to other hazards, such as primarily riverine flooding or wildfires, or to other locations? The only way to answer this question is with more such studies, of more hazards, in more locations. This single-event and single location characteristic of our work can also be perceived as an advantage, as it provides a specific causal link between greenhouse gas emissions and very specific harms. Indeed, we identify harms to specific residential properties. As noted in a recent analysis of the role of event attribution and the law, “one critical question for courts…is to what extent observational evidence of local impacts…can be used to support claims of injury in the absence of an attribution study of a matching geographic and temporal scope showing that the observed impact was caused by anthropogenic influence on climate change” (ref. ^44^, p. 235). Our study provides exactly this missing link, in as much as it “supports claims of injury” for specific properties, after a specific event. As such, our study does not only document the inequalities associated with climate change impacts. In the methodology we developed, it also provides a tool that can assist in redressing these inequalities by assigning clearer chains of causality, and consequently, liability.

Notwithstanding this local relevance of our findings, understanding the climate change justice challenges for other places and other hazards is essential not only for building the climate justice-focused multidisciplinary research synthesis outlined here but also for documenting and attenuating the inequalities in climate change-attributed impacts in marginalized communities worldwide.

## Methods

### Data

The empirical analysis we undertake in this study is based on combining information from geospatial data from five different sources. Geospatial data were analyzed in ArcGISPro 2.9.2 and QGIS 3.16.

First, data on climate change-attributed flooding comes from Wehner and Sampson’s (2021) climate change-attribution hydrodynamic models^16^. To determine the effect of climate change on this baseline flood, seven scenarios of the percentage increase in precipitation based on peer-reviewed research were used to calculate the spatial extent of flooding: 7% (the lowest precipitation change-attribution level as set by the Clausius–Clapeyron scaling as noted by ref. ^10^); 8% (the lower bound of ref. ^12^); 13% (the lower bound of ref. ^13^); 19% (the likely lower bound of the small region of ref. ^10^ and the upper bound of ref. ^12^); 20% (best estimate by ref. ^13^); 24% (best estimate of the large region by ref. ^10^), and 38% (the best estimate of the small region by ref. ^10^ and near the upper bound of the estimate by ref. ^13^).

In short, these flood maps estimate the counterfactual flood impacts from Hurricane Harvey if the identified climate change-attributed rainfall (as a percent of total rainfall) did not occur. Each of these climate change-attribution studies used different modeling techniques to model how climate change increased precipitation but all align in tracing how the storm’s rainfall differed from historical averages due to biophysical changes; especially the increased moisture content of the air and increased temperature of the Gulf of Mexico at the time of the storm. The full hydrological model used to construct the data built on these scenarios we utilize here is described in refs. ^20,45^.

Second, building data is sourced from the National Structure Inventory (NSI) of the U.S. Army Corps of Engineers, and represents every structure in Harris County as a point. The NSI was constructed by combining information from many datasets—including Census data, Microsoft building footprints, CoreLogic parcels, and ESRI business layers—to produce the most accurate possible inventory for assessing natural hazard risk^45^. The data includes information on occupancy type, first-floor elevation (including foundation type and whether the structure was built before or after the area was mapped as part of the 100-year floodplain), and the replacement value in order to link them to depth-damage functions and assess their flood vulnerability. We only include structures flooded above a 20 cm threshold; as very shallow flood depths are unlikely to cause much damage from surface water or pluvial flooding. Since it is composed of proprietary data, this dataset is not publicly available. Further details can be found in refs. ^20,46^. Our damage estimates are somewhat lower than another recent study: we estimate residential damages at $6.42 billion compared to $11.1 billion in ref. ^47^. We attribute these differences primarily to our calculation of building damages using replacement value instead of market value (as the latter also includes the value of the land), as well as possible differences in the depth-damage functions we use, in the modeling of riverine networks, and in the study’s spatial domain. Further, we believe our use of replacement value instead of market value as a depth-damage function is justified for a few reasons. First, replacement value directly estimates impacts on residential structures and contents from flooding. This differs from approaches using a market value which includes the land value that has characteristics (such as neighborhood desirability or how the land use of the building affects the value of the land) that are not easily measured. While economic models exist that could describe the damage as a function of market value such as hedonic property value modeling for cost-benefit analyses relating to environmental impacts^48^, our use of depth-damage functions is favored because of the closer focus on the replacement value of estimated damages. Second, there are large disparities in market value based on the racial composition of neighborhoods^21,22^ meaning that a depth-damage function for market value could undercount damages in minority and/or low-income neighborhoods. Still, future research might consider other approaches such as hedonic models that take into account market value because the present approach with replacement value does not measure the land value of the parcel or how the land value of the parcel may be affected by damages to the buildings in the parcel.

Third, parcel-level data is obtained from the 2016 Harris County Appraisal District (HCAD) database. Harris County is the central county of the Houston metropolitan area. These data include more than 1.4 million parcels and are updated annually. Our study focuses only on the 1.1 million parcels which include residential property. We do not focus on commercial buildings as they are more variable in their structural vulnerabilities and their financial value, making the modeling of damage considerably more difficult. Building data is merged with the parcel data using a spatial join in GIS software. Among residential parcels that had flooded buildings, 7.7% (8822 parcels) had multiple buildings flooded. In these cases, the depths of flooded buildings were averaged, and the damages were summed for the whole parcel.

Fourth, census tract-level data is from the five-year pooled estimates from the 2012–2016 American Community Survey (ACS). The pooled 5-year estimates are used to improve the reliability of the survey. Census tracts are units commonly used in socio-economic geospatial research to denote neighborhoods and have ~4000 residents. We obtain social and demographic data from the ACS on 798 census tracts. Three census tracts had missing values on median income, but these three tracts have only 11 parcels between them; these parcels are dropped from the study sample.

Fifth, data on FEMA-delineated floodplains is obtained for the 100-year floodplain from 2017. This area signifies places that would experience flood inundation in a flood event that has a 1% chance of occurring in a given year. Data were obtained from the Urban Data Platform of Rice University’s Kinder Institute for Urban Research^49^.

### Measurements and calculations

The flood maps generated with the first dataset were intersected with data on the residential buildings in Harris County from the second dataset. Climate change-attributed depths and damages are calculated using the baseline flood scenarios described above. For each scenario, both the flooding depth and damages are subtracted from the baseline (the actual flooding that occurred) to determine the amount of flooding (in terms of depth or damages) that could be attributed to climate change. Each scenario produces two variables from these calculations: attributable flood depths (with depths below 20 cm set to 0; see above) and damages to every building.

The second set of variables is then generated indicating if the parcel’s buildings did or did not flood during Hurricane Harvey because of climate change. If a parcel had $0 flood damage or depths below 20 cm in a given counterfactual scenario but had flood damages or depths in the baseline (actual) scenario, then the parcel was assigned a value of 1 to denote that it flooded only because of the presence of climate change-attributed precipitation. Cases that did not flood at all were coded as 0, and cases that would have flooded regardless of climate change-attributed flood depths or damages are assigned as “anyway flooded” and are excluded from the analysis that focuses only on parcels that would not have flooded without climate change.

Census tract-level variables include racial composition and median income. Racial composition is measured by three variables: (1) proportion Hispanic or Latino (termed here as Latina/x/o; see ref. ^50^), (2) proportion non-Latina/x/o black, and (3) proportion non-Latina/x/o, non-black, and non-white (i.e., American Indian or Alaska Native, Asian, two or more races, or another race). The proportion of non-Latina/x/o white is the reference group with which we compare the other ethnic/racial composition measures.

The median income is measured as the median income of households in the census tract in the previous 12 months and is measured in 2016 inflation-adjusted U.S. dollars. The median income is divided by 10,000 to improve the interpretability of regression coefficients. Additionally, to test for potential intersections between race and class, we employ moderating effects with interaction terms between median income and each of the three racial composition variables.

We use four parcel-level variables. First, the appraised value is the full value of the parcel including the parcel’s building, land, agricultural value, and any value of extra features. This variable is also divided by 10,000 to improve the interpretability of regression coefficients. In the estimated econometric models, a squared term for appraised value is included as some previous research indicates evidence for a nonlinear (concave) wealth effect (wealth here is proxied by the appraised value of the parcel). Second, a categorical variable denoting whether the parcel is a single-family residential parcel (the reference category), a mobile home, or a multifamily parcel. Multifamily residential parcels include apartment-style condominiums, two-family homes, three-family homes, and multifamily homes. Third, we measure the year the structure was built. In a small number of cases where there are two or more residential structures in a parcel, we assign this value as the earliest built structure in the parcel. Fourth, we measure floodplain location with a binary variable denoting if a building in the parcel is located in the FEMA 100-year floodplain by conducting an intersect in GIS software between the FEMA 100-year floodplain and all buildings in Harris County, Texas.

### Analytical and estimation strategy

We first present descriptive statistics about depths and damages relating to climate change-attributed flooding during Hurricane Harvey. All findings are presented at the parcel-level. Analyses were conducted in Stata 16.1.

We next estimate the following equation:

1 $${Y}_{{ic}}^{{{{{{{\mathrm{scn}}}}}}}}=\alpha +{\beta }^{1}{X}_{c}+{\beta }^{2}{V}_{{ic}}+{\varepsilon }_{i}$$

With ${Y}_{i}^{{{{{{{\mathrm{scn}}}}}}}}$ denoting either the depth of flooding attributed to climate change in parcel *i* and in census tract *c*, or the amount of damage attributed to climate change from this flooding (calculated using the damage functions described above). Each one of these is estimated for each climate change scenario (scn: from 7 to 38% less precipitation without climate change). *X*_*c*_ is the vector of variables denoting the composition of the census tract in which the parcel is located (the ethnic composition variables and the median income. *V*_*ic*_ is a vector of measures associated with each specific parcel (the appraised value of the parcel, and whether it is a single-family home, mobile home, or multifamily home). In some specifications, we also interact with some of the *X*_*c*_ and *V*_*ic*_ variables. The *β* coefficients denote the association of these measures with the climate change attributable impact on these properties.

Since both dependent variables (attributable depth and damage) are censored on the left at zero, we estimate these with a Tobit regression model (the results are reported in the Article and Supplementary Information). The error term (*ε*_*i*_) is assumed to be independently and identically distributed, but there still could be collinearities among parcels within a census tract. We, therefore, cluster the standard errors at the census tract (*c*) level to account for any unmeasured similarity that these within-tract parcels may have compared to parcels elsewhere in the county.

In addition to these Tobit regression models as specified in Eq. 1, we also estimated a binary logistic regression model predicting whether the parcel flooded because of climate change (or would have otherwise not been flooded).

2 $${{{{{{{\mathrm{FL}}}}}}}}_{i}^{{{{{{{\mathrm{scn}}}}}}}}=\frac{{e}^{\alpha +{\beta }^{1}{X}_{c}+{\beta }^{2}{V}_{i}+{\varepsilon }_{i}}}{1+{e}^{\alpha +{\beta }^{1}{X}_{c}+{\beta }^{2}{V}_{i}+{\varepsilon }_{i}}}$$

In this case, the dependent variable (${{{{{{{\mathrm{FL}}}}}}}}_{i}^{{{{{{{\mathrm{scn}}}}}}}}$) is a binary indicator (=1) noting that the property had a flood depth of >20 cm and had also less than that for the scenario (scn) being assessed. Parcels that always had flood depth <20 cm in both the scenario being assessed and the actual flood are the default category (=0). Parcels that had depths >20 cm in both scenarios are excluded from this analysis to focus only on parcels that would not have flooded in the no climate change scenarios.

We use similarly clustered standard errors at census tracts as in the Tobit regression model (Eq. 1). The odds ratios estimated from the logit estimation are presented in the tables of results. In estimating Eq. 2, the sample is smaller than Eq. 1 since properties that would have anyway been flooded, even without the additional precipitation attributed to climate change, are excluded. As such, the sample includes 793 to 795 census tracts as in some scenarios a few census tracts no longer had any valid observations (i.e., all parcels had flood depths/damages even with the reduced modeled precipitation).

### Reporting summary

Further information on research design is available in the Nature Research Reporting Summary linked to this article.

## Supplementary information

Supplementary Information

Reporting Summary

## Data Availability

The data that support the findings of this study on flood area and volume are available at: https://portal.nersc.gov/cascade/Harvey/. Data on flood depths and damages at the building level are available from Fathom but restrictions apply to the availability of these data, which were used under license for the current study, and so are not publicly available. The data were available for non-commercial academic research upon reasonable request from Fathom. Data on parcels are available from the Harris County Appraisal District: https://hcad.org/. Data on neighborhood socio-demographics are available from the National Historical Geographic Information Systems: https://www.nhgis.org/. Data on FEMA floodplain location are available from the Kinder Institute for Urban Research at Rice University: https://www.kinderudp.org/#/datasetCatalog/5je3glm092ky.
