# Supplementary material for: Social inequalities in climate change-attributed impacts of Hurricane Harvey
Source: Nat Commun. 2022 Aug 25;13:3418. doi: 10.1038/s41467-022-31056-2 (PMC9411551; doi:10.1038/s41467-022-31056-2)
Supplement: Supplementary file 1 — Supplementary Information [file 41467_2022_31056_MOESM1_ESM.pdf]

Supplementary Information for "Social Inequalities in Climate Change-Attributed Impacts of Hurricane Harvey"

Kevin T. Smiley, Ilan Noy, Michael F. Wehner, David J. Frame, Christopher C. Sampson, and Oliver E. J. Wing.

Table of Contents

1) Table S1: Summary Statistics for Flood Depths (in m) Because of Climate Change-Attributed Flooding For Parcels during Hurricane Harvey in Harris County, TX

2) Table S2: Summary Statistics for Flood Damages (in US $) Because of Climate Change-Attributed Flooding For Parcels during Hurricane Harvey in Harris County, TX

3) Table S3: Summary Statistics for Binary Variables of Whether Parcel Would Have Flood Depths or Flood Damages Only Because of Climate Change-Attributed Flooding during Hurricane Harvey in Harris County, TX

4) Table S4: Summary Statistics for Parcel-Level Variables in Harris County, TX

5) Table S5: Summary Statistics for Census Tract-Level Variables in Harris County, TX

6) Table S6: Tobit Regression of Climate Change-Attributed Depths for Hurricane Harvey in Harris County, Texas

7) Table S7: Tobit Regression of Climate Change-Attributed Damages for Hurricane Harvey in Harris County, Texas

8) Table S8: Logistic Regression of Whether Parcel Had Flood Depths Because of Climate Change for Hurricane Harvey in Harris County, Texas

9) Table S9: Logistic Regression of Whether Parcel Had Flood Damages Because of Climate Change for Hurricane Harvey in Harris County, Texas

10) Table S10: Tobit Regression Including Moderating Relationship for Floodplain Location of Climate Change-Attributed Depths

for Hurricane Harvey in Harris County, Texas

11) Table S11: Tobit Regression Including Moderating Relationship for Floodplain Location of Climate Change-Attributed Damages for Hurricane Harvey in Harris County, Texas

12) Table S12: Logistic Regression Including Moderating Relationship for Floodplain Location of Whether Parcel Had Flood Depths Because of Climate Change for Hurricane Harvey in Harris County, Texas

13) Table S13: Logistic Regression Including Moderating Relationship for Floodplain Location of Whether Parcel Had Flood Damages Because of Climate Change for Hurricane Harvey in Harris County, Texas

Table S1. Summary Statistics for Flood Depths (in m) Because of Climate Change-Attributed Flooding For Parcels during Hurricane Harvey in Harris County, TX

Summary Statistics for All Parcels

	mean	sd
7% Scenario	0.010	0.037
8% Scenario	0.011	0.042
13% Scenario	0.017	0.066
19% Scenario	0.023	0.092
20% Scenario	0.024	0.096
24% Scenario	0.027	0.111
38% Scenario	0.037	0.157
Observations	1108198	

Summary Statistics For Only Flooded Parcels

	mean	sd
7% Scenario	0.103	0.072
8% Scenario	0.116	0.082
13% Scenario	0.177	0.132
19% Scenario	0.241	0.188
20% Scenario	0.251	0.197
24% Scenario	0.287	0.233
38% Scenario	0.390	0.346
Observations	106045	

Table S2. Summary Statistics for Flood Damages (in US $) Because of Climate Change-Attributed Flooding For Parcels during Hurricane Harvey in Harris County, TX

Summary Statistics for All Parcels

	mean	sd
7% Scenario	962.580	6189.494
8% Scenario	1255.999	7595.468
13% Scenario	1708.558	10670.855
19% Scenario	2070.789	13265.558
20% Scenario	2153.770	13829.954
24% Scenario	2508.776	16118.812
38% Scenario	3341.966	21115.825
Observations	1108198	

Summary Statistics For Only Flooded Parcels

	mean	sd
7% Scenario	10001.492	17539.968
8% Scenario	13050.205	21107.222
13% Scenario	17752.428	29971.709
19% Scenario	21516.114	37550.723
20% Scenario	22378.307	39175.877
24% Scenario	26066.934	45667.220
38% Scenario	34724.022	59524.145
Observations	106657	

Table S3: Summary Statistics for Binary Variables of Whether Parcel Would Have Flood Depths or Flood Damages Only Because of Climate Change-Attributed Flooding during Hurricane Harvey in Harris County, TX

	mean
7% Scenario	0.013
8% Scenario	0.015
13% Scenario	0.023
19% Scenario	0.031
20% Scenario	0.033
24% Scenario	0.037
38% Scenario	0.051

Note: Population does not include parcels that would have flooded without climate change.

Table S4: Summary Statistics for Parcel-Level Variables in Harris County, TX

	mean	sd
Appraised Value (in 10,000s)	25.699	105.832
Single-Family Homes	0.918	-
Mobile Homes	0.01	-
Multi-Family Homes	0.072	-
FEMA 100-Year Floodplain	0.09	-
Year Built	1981.36	22.36
Observations	1108198	

Table S5: Summary Statistics for Census Tract-Level Variables in Harris County, TX

	mean	sd
Prop. Latinx	0.418	0.257
Prop. Black	0.187	0.208
Prop. Other Race	0.080	0.081
Prop. Black	0.187	0.208
Median Income (in 10,000s)	5.646	3.339
Observations	795	

Note: Variables calculated for the population of census tracts.

Table S6: Tobit Regression of Climate Change-Attributed Depths for Hurricane Harvey in Harris County, Texas

	(1)<br>7% Scenario	(2)<br>8% Scenario	(3)<br>13% Scenario	(4)<br>19% Scenario	(5)<br>20% Scenario	(6)<br>24% Scenario	(7)<br>38% Scenario
Mobile Homes	-0.058*	-0.065*	-0.101*	-0.140*	-0.146*	-0.170*	-0.244*
	(0.027)	(0.030)	(0.047)	(0.064)	(0.067)	(0.077)	(0.104)
Multi-Family	-0.114***	-0.130***	-0.200***	-0.274***	-0.286***	-0.329***	-0.455***
	(0.015)	(0.017)	(0.026)	(0.036)	(0.037)	(0.043)	(0.060)
Appraised Value (in 10,000s)	0.000**	0.000*	0.000**	0.000*	0.000*	0.000**	0.000**
	(0.000)	(0.000)	(0.000)	(0.000)	(0.000)	(0.000)	(0.000)
Appraised Value (in 10,000s)*Appraised Value (in 10,000s)	-0.000	-0.000	-0.000	-0.000	-0.000	-0.000	-0.000
	(0.000)	(0.000)	(0.000)	(0.000)	(0.000)	(0.000)	(0.000)
FEMA 100-Year Floodplain	0.118***	0.134***	0.208***	0.287***	0.300***	0.346***	0.484***
	(0.011)	(0.012)	(0.019)	(0.026)	(0.027)	(0.032)	(0.044)
Year Built	-0.001***	-0.002***	-0.003***	-0.004***	-0.004***	-0.004***	-0.006***
	(0.000)	(0.000)	(0.000)	(0.001)	(0.001)	(0.001)	(0.001)
Prop. Latina/x/o	0.336***	0.380***	0.587***	0.803***	0.838***	0.965***	1.341***
	(0.075)	(0.086)	(0.133)	(0.184)	(0.192)	(0.222)	(0.312)
Prop. Black	-0.197	-0.223	-0.344	-0.472	-0.492	-0.568	-0.798
	(0.110)	(0.124)	(0.193)	(0.265)	(0.277)	(0.319)	(0.446)
Prop. Other Race	-0.071	-0.079	-0.121	-0.165	-0.172	-0.199	-0.285
	(0.204)	(0.232)	(0.359)	(0.493)	(0.515)	(0.594)	(0.828)

Median Income (in 10,000s)	0.017*	0.019*	0.030*	0.041*	0.043*	0.049*	0.068*
	(0.007)	(0.008)	(0.013)	(0.018)	(0.018)	(0.021)	(0.030)
Prop. Black*Median Income (in 10,000s)	0.025	0.029	0.043	0.059	0.061	0.070	0.098
	(0.027)	(0.030)	(0.047)	(0.064)	(0.067)	(0.077)	(0.108)
Prop. Latina/x/o*Median Income (in 10,000s)	-0.045*	-0.051*	-0.079*	-0.108*	-0.113*	-0.130*	-0.180*
	(0.019)	(0.022)	(0.034)	(0.047)	(0.049)	(0.057)	(0.079)
Prop. Other Race*Median Income (in 10,000s)	-0.036	-0.041	-0.064	-0.088	-0.092	-0.106	-0.147
	(0.032)	(0.036)	(0.056)	(0.077)	(0.080)	(0.093)	(0.130)
Constant	2.586***	2.922***	4.499***	6.140***	6.402***	7.358***	10.166***
	(0.435)	(0.492)	(0.764)	(1.054)	(1.100)	(1.272)	(1.783)
Observations	1108198	1108198	1108198	1108198	1108198	1108198	1108198

Standard errors in parentheses
Standard errors clustered within census tracts
Statistical tests are two-sided.
* $p < 0.05$, ** $p < 0.01$, *** $p < 0.001$

Table S7: Tobit Regression of Climate Change-Attributed Damages for Hurricane Harvey in Harris County, Texas

	(1)<br>7% Scenario	(2)<br>8% Scenario	(3)<br>13% Scenario	(4)<br>19% Scenario	(5)<br>20% Scenario	(6)<br>24% Scenario	(7)<br>38% Scenario
Mobile Homes	-6698.296	-8515.547	-11155.532	-12728.225	-13974.716	-16833.565	-22485.544
	(3630.754)	(4534.525)	(6511.335)	(8597.070)	(8660.438)	(9950.144)	(12907.822)
Multi-Family	-12359.390***	-15316.817***	-21203.781***	-26077.798***	-27291.861***	-31622.427***	-41537.089***
	(1573.035)	(1983.314)	(2856.506)	(3804.834)	(3751.408)	(4342.615)	(5786.324)
Appraised Value (in 10,000s)	35.427***	44.633***	63.122***	78.977***	78.956***	90.039***	118.819***
	(5.441)	(6.874)	(10.092)	(13.292)	(13.389)	(15.480)	(19.783)
Appraised Value (in 10,000s)*Appraised Value (in 10,000s)	-0.004***	-0.004***	-0.006***	-0.008***	-0.008***	-0.009***	-0.012***
	(0.001)	(0.001)	(0.002)	(0.002)	(0.002)	(0.003)	(0.003)
FEMA 100-Year Floodplain	17082.259***	21421.903***	30577.584***	40521.434***	39172.316***	44540.009***	59673.766***
	(1616.761)	(2024.828)	(3043.331)	(4263.193)	(4115.806)	(4756.561)	(6242.381)
Year Built	-207.333***	-258.542***	-383.949***	-503.162***	-486.906***	-537.867***	-731.664***
	(28.433)	(35.470)	(52.471)	(72.057)	(69.003)	(78.548)	(106.573)
Prop. Latina/x/o	42387.946***	52850.647***	75049.659***	99562.382***	96245.978***	109883.384***	146089.445***
	(9300.629)	(11712.993)	(17131.100)	(23122.930)	(22228.551)	(25333.949)	(33922.639)
Prop. Black	-24300.793	-30278.732	-44152.595	-69941.969*	-55625.818	-60950.022	-83405.492

	(13772.751)	(17340.420)	(25336.610)	(35449.332)	(32584.418)	(36699.222)	(49818.786)
Prop. Other Race	-12556.911	-15787.320	-20358.955	-23562.217	-25886.556	-31731.151	-41412.489
	(25814.291)	(32353.793)	(46833.142)	(63978.912)	(59985.583)	(67689.970)	(91617.440)
Median Income (in 10,000s)	1967.673*	2451.479*	3543.480*	4779.329*	4558.075*	5138.290*	6843.985*
	(856.131)	(1083.616)	(1560.424)	(2090.676)	(2006.640)	(2278.877)	(3075.339)
Prop. Black*Median Income (in 10,000s)	3991.229	4879.881	7008.997	10643.578	8795.172	9682.024	12926.780
	(3352.913)	(4216.529)	(6202.662)	(8676.768)	(7973.184)	(8935.585)	(12139.877)
Prop. Latina/x/o*Median Income (in 10,000s)	-5335.802*	-6635.861*	-9400.997*	-12699.105*	-11922.360*	-13421.244*	-17647.823*
	(2315.980)	(2925.721)	(4244.583)	(5735.129)	(5478.747)	(6221.917)	(8414.736)
Prop. Other Race*Median Income (in 10,000s)	-3755.405	-4763.289	-7280.857	-10809.178	-9267.221	-9936.617	-13570.820
	(3775.627)	(4769.822)	(6904.333)	(9354.725)	(8833.474)	(9959.687)	(13470.497)
Constant	357090.316***	445833.761***	665218.965***	870921.679***	841824.157***	925793.071***	1263358.957***
	(55974.727)	(69959.378)	(102752.410)	(140411.235)	(134170.769)	(152376.862)	(207126.315)
Observations	1108198	1108198	1108198	1108198	1108198	1108198	1108198

Standard errors in parentheses
Standard errors clustered within census tracts
Statistical tests are two-sided.
* $p < 0.05$, ** $p < 0.01$, *** $p < 0.001$

Table S8: Logistic Regression of Whether Parcel Had Flood Depths Because of Climate Change for Hurricane Harvey in Harris County, Texas

	(1)<br>7% Scenario	(2)<br>8% Scenario	(3)<br>13% Scenario	(4)<br>19% Scenario	(5)<br>20% Scenario	(6)<br>24% Scenario	(7)<br>38% Scenario
Mobile Homes	0.747	0.756	0.712	0.676	0.678	0.715	0.701
	(0.248)	(0.253)	(0.232)	(0.224)	(0.222)	(0.250)	(0.252)
Multi-Family	0.295***	0.293***	0.289***	0.286***	0.289***	0.281***	0.283***
	(0.049)	(0.048)	(0.048)	(0.046)	(0.046)	(0.043)	(0.041)
Appraised Value (in 10,000s)	1.001*	1.001*	1.001	1.001	1.001	1.001	1.001
	(0.000)	(0.000)	(0.001)	(0.001)	(0.001)	(0.001)	(0.001)
Appraised Value (in 10,000s)*Appraised Value (in 10,000s)	1.000	1.000	1.000	1.000	1.000	1.000	1.000
	(0.000)	(0.000)	(0.000)	(0.000)	(0.000)	(0.000)	(0.000)
FEMA 100-Year Floodplain	2.917***	2.970***	3.068***	3.244***	3.267***	3.350***	3.553***
	(0.329)	(0.335)	(0.356)	(0.381)	(0.384)	(0.399)	(0.437)
Year Built	0.985***	0.985***	0.984***	0.984***	0.984***	0.984***	0.984***
	(0.002)	(0.002)	(0.002)	(0.002)	(0.002)	(0.002)	(0.002)
Prop. Latina/x/o	17.059***	17.971***	22.304***	21.218***	21.542***	22.110***	22.893***
	(10.213)	(10.767)	(14.029)	(13.444)	(13.672)	(14.200)	(15.104)

Prop. Black	0.097*	0.097*	0.098*	0.103*	0.109	0.125	0.141
	(0.105)	(0.106)	(0.110)	(0.119)	(0.126)	(0.147)	(0.174)
Prop. Other Race	0.172	0.222	0.289	0.331	0.362	0.445	0.610
	(0.289)	(0.364)	(0.492)	(0.587)	(0.646)	(0.809)	(1.189)
Median Income (in 10,000s)	1.137**	1.140**	1.151**	1.156**	1.160**	1.167**	1.170**
	(0.047)	(0.047)	(0.052)	(0.056)	(0.056)	(0.059)	(0.064)
Prop. Black*Median Income (in 10,000s)	1.664*	1.656*	1.625	1.585	1.569	1.514	1.458
	(0.397)	(0.401)	(0.413)	(0.424)	(0.424)	(0.423)	(0.431)
Prop. Latina/x/o*Median Income (in 10,000s)	0.678*	0.671*	0.651**	0.673*	0.675*	0.682*	0.712
	(0.105)	(0.105)	(0.107)	(0.115)	(0.116)	(0.120)	(0.131)
Prop. Other Race*Median Income (in 10,000s)	0.895	0.882	0.855	0.823	0.811	0.789	0.769
	(0.167)	(0.166)	(0.177)	(0.183)	(0.180)	(0.181)	(0.191)
Observations	1015769	1017543	1026002	1034635	1036024	1040998	1055759

Exponentiated coefficients; Standard errors in parentheses
Standard errors clustered within census tracts
Statistical tests are two-sided.
* $p < 0.05$, ** $p < 0.01$, *** $p < 0.001$

Table S9: Logistic Regression of Whether Parcel Had Flood Damages Because of Climate Change for Hurricane Harvey in Harris County, Texas

	(1)<br>7% Scenario	(2)<br>8% Scenario	(3)<br>13% Scenario	(4)<br>19% Scenario	(5)<br>20% Scenario	(6)<br>24% Scenario	(7)<br>38% Scenario
Mobile Homes	0.746	0.755	0.709	0.670	0.673	0.703	0.704
	(0.249)	(0.255)	(0.232)	(0.222)	(0.221)	(0.244)	(0.254)
Multi-Family	0.327***	0.325***	0.322***	0.316***	0.319***	0.311***	0.310***
	(0.053)	(0.051)	(0.052)	(0.049)	(0.049)	(0.047)	(0.043)
Appraised Value (in 10,000s)	1.001*	1.001*	1.001*	1.002*	1.001**	1.001**	1.001**
	(0.001)	(0.001)	(0.001)	(0.001)	(0.001)	(0.001)	(0.001)
Appraised Value (in 10,000s)*Appraised Value (in 10,000s)	1.000	1.000	1.000	1.000	1.000	1.000	1.000
	(0.000)	(0.000)	(0.000)	(0.000)	(0.000)	(0.000)	(0.000)
FEMA 100-Year Floodplain	2.907***	2.965***	3.065***	3.239***	3.262***	3.348***	3.546***
	(0.329)	(0.335)	(0.357)	(0.381)	(0.384)	(0.399)	(0.436)
Year Built	0.984***	0.984***	0.984***	0.984***	0.984***	0.984***	0.984***
	(0.002)	(0.002)	(0.002)	(0.002)	(0.002)	(0.002)	(0.002)

Prop. Latina/x/o	16.392***	17.234***	21.425***	20.472***	20.782***	21.233***	22.090***
	(9.866)	(10.378)	(13.510)	(12.956)	(13.154)	(13.607)	(14.551)
Prop. Black	0.099*	0.099*	0.100*	0.104*	0.110	0.126	0.141
	(0.107)	(0.108)	(0.112)	(0.120)	(0.126)	(0.147)	(0.173)
Prop. Other Race	0.158	0.203	0.262	0.306	0.333	0.408	0.563
	(0.266)	(0.332)	(0.445)	(0.540)	(0.590)	(0.738)	(1.094)
Median Income (in 10,000s)	1.131**	1.133**	1.144**	1.149**	1.153**	1.159**	1.163**
	(0.047)	(0.047)	(0.052)	(0.055)	(0.056)	(0.058)	(0.064)
Prop. Black*Median Income (in 10,000s)	1.663*	1.654*	1.621	1.584	1.569	1.516	1.461
	(0.396)	(0.400)	(0.412)	(0.423)	(0.423)	(0.422)	(0.432)
Prop. Latina/x/o*Median Income (in 10,000s)	0.686*	0.679*	0.658*	0.679*	0.681*	0.689*	0.717
	(0.106)	(0.106)	(0.108)	(0.116)	(0.117)	(0.121)	(0.132)
Prop. Other Race*Median Income (in 10,000s)	0.908	0.896	0.868	0.834	0.822	0.800	0.778
	(0.171)	(0.169)	(0.180)	(0.185)	(0.182)	(0.184)	(0.194)
Observations	1015209	1016988	1025479	1034141	1035536	1040524	1055313

Exponentiated coefficients; Standard errors in parentheses
Standard errors clustered within census tracts
Statistical tests are two-sided.
* $p < 0.05$, ** $p < 0.01$, *** $p < 0.001$

Table S10: Tobit Regression Including Moderating Relationship for Floodplain Location of Climate Change-Attributed Depths for Hurricane Harvey in Harris County, Texas

	(1)<br>7% Scenario	(2)<br>8% Scenario	(3)<br>13% Scenario	(4)<br>19% Scenario	(5)<br>20% Scenario	(6)<br>24% Scenario	(7)<br>38% Scenario
Mobile Homes	-0.057*	-0.065*	-0.100*	-0.138*	-0.145*	-0.168*	-0.241*
	(0.025)	(0.029)	(0.044)	(0.060)	(0.063)	(0.072)	(0.098)
Multi-Family	-0.115***	-0.130***	-0.201***	-0.275***	-0.287***	-0.330***	-0.457***
	(0.014)	(0.016)	(0.025)	(0.035)	(0.036)	(0.042)	(0.059)
Appraised Value (in 10,000s)	0.000*	0.000*	0.000*	0.000*	0.000*	0.000*	0.000*
	(0.000)	(0.000)	(0.000)	(0.000)	(0.000)	(0.000)	(0.000)
Appraised Value (in 10,000s)*Appraised Value (in 10,000s)	-0.000	-0.000	-0.000	-0.000	-0.000	-0.000	-0.000
	(0.000)	(0.000)	(0.000)	(0.000)	(0.000)	(0.000)	(0.000)
FEMA 100-Year Floodplain	0.254**	0.288**	0.449**	0.624**	0.653**	0.755**	1.074**
	(0.082)	(0.093)	(0.144)	(0.198)	(0.207)	(0.239)	(0.334)
Year Built	-0.001***	-0.002***	-0.002***	-0.003***	-0.003***	-0.004***	-0.005***
	(0.000)	(0.000)	(0.000)	(0.001)	(0.001)	(0.001)	(0.001)
Prop. Latina/x/o	0.390***	0.442***	0.682***	0.934***	0.975***	1.123***	1.562***
	(0.081)	(0.092)	(0.142)	(0.196)	(0.205)	(0.237)	(0.333)

Prop. Black	-0.217	-0.246	-0.378	-0.516	-0.537	-0.619	-0.860
	(0.127)	(0.143)	(0.222)	(0.306)	(0.319)	(0.369)	(0.514)
Prop. Other Race	-0.031	-0.034	-0.049	-0.061	-0.063	-0.069	-0.087
	(0.262)	(0.297)	(0.459)	(0.630)	(0.658)	(0.758)	(1.055)
Median Income (in 10,000s)	0.019*	0.022*	0.034*	0.046*	0.048*	0.056*	0.077*
	(0.008)	(0.009)	(0.015)	(0.020)	(0.021)	(0.024)	(0.034)
Prop. Black*Median Income (in 10,000s)	0.028	0.031	0.047	0.064	0.066	0.076	0.105
	(0.030)	(0.034)	(0.053)	(0.073)	(0.076)	(0.088)	(0.122)
FEMA 100-Year Floodplain*Prop. Black	0.067	0.074	0.106	0.132	0.135	0.150	0.166
	(0.154)	(0.175)	(0.271)	(0.373)	(0.389)	(0.449)	(0.626)
FEMA 100-Year Floodplain*Median Income (in 10,000s)	-0.011	-0.012	-0.019	-0.026	-0.028	-0.032	-0.044
	(0.009)	(0.010)	(0.015)	(0.021)	(0.022)	(0.026)	(0.035)

FEMA 100-Year Floodplain*Prop. Black*Median Income (in 10,000s)	0.002	0.002	0.005	0.008	0.009	0.011	0.018
	(0.036)	(0.041)	(0.063)	(0.086)	(0.090)	(0.104)	(0.144)
Prop. Latina/x/o*Median Income (in 10,000s)	-0.049*	-0.056*	-0.086*	-0.117*	-0.122*	-0.140*	-0.193*
	(0.021)	(0.023)	(0.036)	(0.050)	(0.052)	(0.060)	(0.084)
FEMA 100-Year Floodplain*Prop. Latina/x/o	-0.374***	-0.423***	-0.651***	-0.892***	-0.929***	-1.068***	-1.471***
	(0.101)	(0.114)	(0.177)	(0.243)	(0.254)	(0.293)	(0.408)
FEMA 100-Year Floodplain*Prop. Latina/x/o*Median Income (in 10,000s)	0.035	0.039	0.059	0.079	0.081	0.092	0.119
	(0.024)	(0.027)	(0.041)	(0.057)	(0.059)	(0.068)	(0.095)
Prop. Other Race*Median Income (in 10,000s)	-0.046	-0.052	-0.081	-0.112	-0.117	-0.134	-0.187
	(0.044)	(0.050)	(0.078)	(0.107)	(0.111)	(0.129)	(0.179)

FEMA 100-Year Floodplain*Prop. Other Race	-0.040	-0.047	-0.076	-0.124	-0.132	-0.159	-0.276
	(0.297)	(0.337)	(0.521)	(0.716)	(0.747)	(0.862)	(1.196)
FEMA 100-Year Floodplain*Prop. Other Race*Median Income (in 10,000s)	0.022	0.025	0.037	0.052	0.054	0.061	0.084
	(0.044)	(0.050)	(0.077)	(0.106)	(0.110)	(0.128)	(0.177)
Constant	2.361***	2.668***	4.105***	5.598***	5.837***	6.706***	9.263***
	(0.429)	(0.486)	(0.755)	(1.039)	(1.085)	(1.253)	(1.755)
Observations	1108198	1108198	1108198	1108198	1108198	1108198	1108198

Standard errors in parentheses
Standard errors clustered within census tracts
Statistical tests are two-sided.
* $p < 0.05$, ** $p < 0.01$, *** $p < 0.001$

Table S11: Tobit Regression Including Moderating Relationship for Floodplain Location of Climate Change-Attributed Damages for Hurricane Harvey in Harris County, Texas

	(1) 7% Scenario	(2) 8% Scenario	(3) 13% Scenario	(4) 19% Scenario	(5) 20% Scenario	(6) 24% Scenario	(7) 38% Scenario
Mobile Homes	-6634.345	-8429.833*	-11046.431	-12617.766	-13843.057	-16678.834	-22272.847
	(3428.393)	(4282.246)	(6143.045)	(8077.263)	(8189.268)	(9420.224)	(12189.978)
Multi-Family	-12441.166***	-15416.451***	-21335.042***	-26256.616***	-27457.907***	-31808.391***	-41797.901***
	(1537.313)	(1939.486)	(2790.725)	(3719.843)	(3667.918)	(4250.053)	(5659.367)
Appraised Value (in 10,000s)	35.378***	44.554***	63.040***	78.975***	78.825***	89.831***	118.542***
	(5.434)	(6.863)	(10.082)	(13.291)	(13.376)	(15.458)	(19.744)
Appraised Value (in 10,000s)*Appraised Value (in 10,000s)	-0.004***	-0.004***	-0.006***	-0.008***	-0.008***	-0.009***	-0.012***
	(0.001)	(0.001)	(0.002)	(0.002)	(0.002)	(0.003)	(0.003)
FEMA 100-Year Floodplain	32055.060**	40953.541**	57370.131**	75573.378**	73501.416**	84058.587**	115580.498**
	(10763.417)	(13444.128)	(19452.280)	(26051.709)	(25142.223)	(28689.361)	(38653.150)
Year Built	-195.938***	-244.205***	-362.896***	-472.887***	-459.601***	-507.000***	-689.408***
	(28.082)	(35.016)	(51.706)	(70.899)	(67.797)	(77.035)	(104.382)
Prop. Latina/x/o	48833.877***	60994.424***	86688.810***	115775.624***	111108.973***	126703.051***	169136.704***
	(10136.800)	(12725.839)	(18722.744)	(25489.385)	(24317.228)	(27638.719)	(36996.857)
Prop. Black	-27819.382	-34416.150	-51019.089	-84058.965*	-64491.413	-69879.155	-95453.587

	(16027.992)	(20140.780)	(29574.005)	(42011.847)	(38066.319)	(42728.230)	(58139.412)
Prop. Other Race	-8648.995	-10774.061	-14113.105	-15758.798	-18417.833	-23063.239	-27523.344
	(33418.314)	(41725.840)	(61095.933)	(85809.398)	(78169.848)	(87174.376)	(118778.654)
Median Income (in 10,000s)	2229.482*	2784.049*	4016.434*	5427.940*	5164.156*	5818.291*	7808.650*
	(998.133)	(1258.107)	(1827.329)	(2483.448)	(2349.294)	(2650.723)	(3584.028)
Prop. Black*Median Income (in 10,000s)	4484.813	5454.948	7955.442	12630.061	9975.672	10826.282	14462.007
	(3817.359)	(4799.256)	(7082.896)	(10000.599)	(9111.245)	(10192.542)	(13881.671)
FEMA 100-Year Floodplain*Prop. Black	13280.426	15238.524	25242.238	47600.971	31786.096	31853.725	40491.626
	(20378.410)	(25563.077)	(37352.724)	(51826.985)	(48134.859)	(54299.420)	(73844.939)
FEMA 100-Year Floodplain*Median Income (in 10,000s)	-1283.500	-1651.403	-2353.049	-3223.927	-3066.702	-3457.049	-4858.782
	(1158.181)	(1440.010)	(2104.801)	(2837.711)	(2720.859)	(3085.219)	(4165.181)
FEMA 100-Year Floodplain*Prop. Black*Median Income (in 10,000s)	-997.431	-1015.030	-1862.886	-4582.871	-2013.846	-1426.994	-1415.137
	(4747.668)	(5951.383)	(8843.537)	(12400.316)	(11402.184)	(12688.248)	(17344.130)

Prop. Latina/x/o*Median Income (in 10,000s)	-5818.155*	-7223.384*	-10285.433*	-14018.665*	-13051.750*	-14663.079*	-19275.457*
	(2481.527)	(3131.216)	(4551.536)	(6166.564)	(5869.197)	(6654.314)	(9009.312)
FEMA 100-Year Floodplain*Prop. Latina/x/o	-43362.175**	-54604.252**	-78352.880**	-106886.627**	-100488.297**	-114109.425**	-154505.654**
	(13434.264)	(16728.549)	(24295.795)	(32598.570)	(31481.449)	(35835.918)	(47856.146)
FEMA 100-Year Floodplain*Prop. Latina/x/o*Median Income (in 10,000s)	4069.042	4987.384	7488.685	10652.625	9603.312	10669.663	13909.161
	(3068.450)	(3827.776)	(5524.192)	(7395.457)	(7109.144)	(8076.724)	(10853.462)
Prop. Other Race*Median Income (in 10,000s)	-5142.990	-6455.778	-9738.580	-14277.755	-12399.486	-13380.886	-18489.697
	(5333.735)	(6712.835)	(9857.464)	(13776.727)	(12615.052)	(14039.122)	(19143.960)
FEMA 100-Year Floodplain*Prop. Other Race	1648.439	404.352	3931.935	7294.994	5693.726	5452.287	-218.882
	(39651.035)	(49262.844)	(72045.699)	(99441.155)	(92442.592)	(104084.173)	(140936.465)
FEMA 100-Year Floodplain*Prop. Other Race*Median Income (in 10,000s)	2867.643	3628.875	5230.094	7431.601	6882.123	7575.673	11076.644
	(5655.730)	(7054.153)	(10441.300)	(14521.100)	(13408.582)	(14980.517)	(20373.201)

Constant	331862.532***	413990.921***	618822.647***	804814.943***	781808.040***	857848.728***	1169960.341***
	(55295.642)	(69086.435)	(101291.554)	(138208.428)	(131867.148)	(149480.349)	(202916.309)
Observations	1108198	1108198	1108198	1108198	1108198	1108198	1108198

Standard errors in parentheses
Standard errors clustered within census tracts
Statistical tests are two-sided.
* $p < 0.05$, ** $p < 0.01$, *** $p < 0.001$

Table S12: Logistic Regression Including Moderating Relationship for Floodplain Location of Whether Parcel Had Flood Depths Because of Climate Change for Hurricane Harvey in Harris County, Texas

	(1)<br>7% Scenario	(2)<br>8% Scenario	(3)<br>13% Scenario	(4)<br>19% Scenario	(5)<br>20% Scenario	(6)<br>24% Scenario	(7)<br>38% Scenario
Mobile Homes	0.786	0.795	0.749	0.710	0.712	0.749	0.731
	(0.254)	(0.260)	(0.236)	(0.225)	(0.223)	(0.249)	(0.248)
Multi-Family	0.296***	0.293***	0.290***	0.287***	0.290***	0.282***	0.284***
	(0.049)	(0.048)	(0.048)	(0.046)	(0.045)	(0.043)	(0.040)
Appraised Value (in 10,000s)	1.001*	1.001*	1.001*	1.001	1.001	1.001	1.001
	(0.000)	(0.000)	(0.001)	(0.001)	(0.001)	(0.001)	(0.001)
Appraised Value (in 10,000s)*Appraised Value (in 10,000s)	1.000	1.000	1.000	1.000	1.000	1.000	1.000
	(0.000)	(0.000)	(0.000)	(0.000)	(0.000)	(0.000)	(0.000)
FEMA 100-Year Floodplain	3.451	3.586	3.279	3.557	3.584	4.065	5.556
	(2.848)	(2.929)	(2.806)	(3.101)	(3.135)	(3.656)	(5.371)
Year Built	0.985***	0.985***	0.985***	0.985***	0.985***	0.985***	0.985***
	(0.002)	(0.002)	(0.002)	(0.002)	(0.002)	(0.002)	(0.002)
Prop. Latina/x/o	24.882***	26.018***	32.156***	29.959***	30.666***	31.871***	34.501***
	(16.976)	(17.738)	(23.051)	(21.767)	(22.308)	(23.462)	(26.327)

Prop. Black	0.059*	0.060*	0.060*	0.061*	0.062*	0.069	0.077
	(0.077)	(0.078)	(0.080)	(0.085)	(0.086)	(0.099)	(0.117)
Prop. Other Race	0.174	0.193	0.294	0.241	0.266	0.274	0.443
	(0.491)	(0.537)	(0.833)	(0.702)	(0.776)	(0.799)	(1.356)
Median Income (in 10,000s)	1.150*	1.152*	1.165*	1.167*	1.172*	1.180*	1.192*
	(0.064)	(0.065)	(0.072)	(0.078)	(0.078)	(0.081)	(0.089)
Prop. Black*Median Income (in 10,000s)	1.798*	1.786*	1.746	1.694	1.686	1.630	1.559
	(0.482)	(0.488)	(0.503)	(0.523)	(0.527)	(0.531)	(0.550)
FEMA 100-Year Floodplain*Prop. Black	9.246	8.425	8.365	7.327	8.261	8.139	6.170
	(16.034)	(14.641)	(15.209)	(13.698)	(15.468)	(15.417)	(12.429)
FEMA 100-Year Floodplain*Median Income (in 10,000s)	1.012	1.010	1.017	1.015	1.011	0.998	0.970
	(0.080)	(0.079)	(0.089)	(0.091)	(0.091)	(0.094)	(0.102)
FEMA 100-Year Floodplain*Prop. Black*Median Income (in 10,000s)	0.739	0.749	0.787	0.851	0.834	0.844	0.920
	(0.272)	(0.279)	(0.313)	(0.355)	(0.349)	(0.359)	(0.419)

Prop. Latina/x/o*Median Income (in 10,000s)	0.664*	0.658*	0.639*	0.665*	0.666*	0.673*	0.704
	(0.111)	(0.112)	(0.114)	(0.125)	(0.125)	(0.130)	(0.142)
FEMA 100-Year Floodplain*Prop. Latina/x/o	0.103*	0.102*	0.106*	0.115*	0.109*	0.094*	0.079*
	(0.109)	(0.107)	(0.117)	(0.126)	(0.120)	(0.104)	(0.090)
FEMA 100-Year Floodplain*Prop. Latina/x/o*Median Income (in 10,000s)	1.251	1.244	1.254	1.198	1.220	1.224	1.198
	(0.313)	(0.311)	(0.327)	(0.315)	(0.319)	(0.317)	(0.310)
Prop. Other Race*Median Income (in 10,000s)	0.791	0.790	0.735	0.731	0.717	0.701	0.652
	(0.304)	(0.307)	(0.301)	(0.311)	(0.306)	(0.305)	(0.298)
FEMA 100-Year Floodplain*Prop. Other Race	26.134	33.277	34.216	50.246	46.737	57.100	31.235
	(86.093)	(107.625)	(113.224)	(169.258)	(157.532)	(194.767)	(113.836)
FEMA 100-Year Floodplain*Prop. Other Race*Median Income (in 10,000s)	0.883	0.873	0.894	0.877	0.892	0.915	1.045
	(0.380)	(0.377)	(0.414)	(0.418)	(0.425)	(0.447)	(0.551)

Observations	1015769	1017543	1026002	1034635	1036024	1040998	1055759

Exponentiated coefficients; Standard errors in parentheses
Standard errors clustered within census tracts
Statistical tests are two-sided.
* $p < 0.05$, ** $p < 0.01$, *** $p < 0.001$

Table S13: Logistic Regression Including Moderating Relationship for Floodplain Location of Whether Parcel Had Flood Damages Because of Climate Change for Hurricane Harvey in Harris County, Texas

	(1)<br>7% Scenario	(2)<br>8% Scenario	(3)<br>13% Scenario	(4)<br>19% Scenario	(5)<br>20% Scenario	(6)<br>24% Scenario	(7)<br>38% Scenario
Mobile Homes	0.786	0.795	0.747	0.704	0.706	0.737	0.735
	(0.256)	(0.261)	(0.236)	(0.224)	(0.222)	(0.244)	(0.249)
Multi-Family	0.327***	0.325***	0.323***	0.317***	0.320***	0.312***	0.311***
	(0.052)	(0.051)	(0.052)	(0.049)	(0.048)	(0.046)	(0.042)
Appraised Value (in 10,000s)	1.002**	1.002*	1.002*	1.002**	1.002**	1.001**	1.001**
	(0.001)	(0.001)	(0.001)	(0.001)	(0.001)	(0.001)	(0.001)
Appraised Value (in 10,000s)*Appraised Value (in 10,000s)	1.000	1.000	1.000	1.000	1.000	1.000	1.000
	(0.000)	(0.000)	(0.000)	(0.000)	(0.000)	(0.000)	(0.000)
FEMA 100-Year Floodplain	3.296	3.429	3.167	3.421	3.433	3.910	5.353
	(2.707)	(2.788)	(2.695)	(2.964)	(2.986)	(3.498)	(5.158)
Year Built	0.985***	0.985***	0.985***	0.985***	0.985***	0.985***	0.985***
	(0.002)	(0.002)	(0.002)	(0.002)	(0.002)	(0.002)	(0.002)
Prop. Latina/x/o	23.860***	24.856***	30.733***	28.802***	29.443***	30.439***	33.057***
	(16.350)	(17.022)	(22.077)	(20.884)	(21.339)	(22.336)	(25.158)

Prop. Black	0.059*	0.059*	0.059*	0.060*	0.061*	0.068	0.076
	(0.076)	(0.077)	(0.079)	(0.083)	(0.085)	(0.097)	(0.116)
Prop. Other Race	0.153	0.166	0.253	0.217	0.238	0.245	0.407
	(0.431)	(0.463)	(0.717)	(0.630)	(0.687)	(0.711)	(1.235)
Median Income (in 10,000s)	1.143*	1.144*	1.157*	1.159*	1.164*	1.172*	1.184*
	(0.065)	(0.065)	(0.072)	(0.077)	(0.077)	(0.080)	(0.088)
Prop. Black*Median Income (in 10,000s)	1.806*	1.795*	1.752	1.701	1.692	1.638	1.564
	(0.484)	(0.489)	(0.504)	(0.523)	(0.527)	(0.532)	(0.551)
FEMA 100-Year Floodplain*Prop. Black	10.809	9.878	9.678	8.227	9.237	8.962	6.376
	(18.626)	(17.038)	(17.539)	(15.344)	(17.257)	(16.937)	(12.837)
FEMA 100-Year Floodplain*Median Income (in 10,000s)	1.017	1.015	1.022	1.020	1.016	1.003	0.974
	(0.079)	(0.078)	(0.088)	(0.091)	(0.091)	(0.094)	(0.102)
FEMA 100-Year Floodplain*Prop. Black*Median Income (in 10,000s)	0.713	0.724	0.761	0.829	0.814	0.825	0.915
	(0.261)	(0.268)	(0.302)	(0.345)	(0.340)	(0.350)	(0.416)

Prop. Latina/x/o*Median Income (in 10,000s)	0.671*	0.666*	0.645*	0.671*	0.672*	0.680*	0.710
	(0.113)	(0.113)	(0.116)	(0.126)	(0.126)	(0.131)	(0.143)
FEMA 100-Year Floodplain*Prop. Latina/x/o	0.100*	0.101*	0.107*	0.116*	0.111*	0.097*	0.082*
	(0.106)	(0.105)	(0.117)	(0.127)	(0.122)	(0.107)	(0.093)
FEMA 100-Year Floodplain*Prop. Latina/x/o*Median Income (in 10,000s)	1.267	1.255	1.257	1.199	1.219	1.221	1.193
	(0.316)	(0.313)	(0.327)	(0.315)	(0.318)	(0.316)	(0.308)
Prop. Other Race*Median Income (in 10,000s)	0.806	0.806	0.750	0.743	0.730	0.713	0.661
	(0.310)	(0.314)	(0.307)	(0.315)	(0.310)	(0.308)	(0.301)
FEMA 100-Year Floodplain*Prop. Other Race	31.624	40.764	40.432	57.408	54.267	64.817	34.057
	(103.772)	(131.432)	(133.145)	(192.307)	(181.803)	(219.735)	(123.485)
FEMA 100-Year Floodplain*Prop. Other Race*Median Income (in 10,000s)	0.864	0.854	0.876	0.861	0.874	0.899	1.030
	(0.371)	(0.368)	(0.405)	(0.408)	(0.414)	(0.437)	(0.541)

Observations	1015209	1016988	1025479	1034141	1035536	1040524	1055313

Exponentiated coefficients; Standard errors in parentheses
Standard errors clustered within census tracts
Statistical tests are two-sided.
* $p < 0.05$, ** $p < 0.01$, *** $p < 0.001$
