# Supplementary material for: Social inequalities in climate change-attributed impacts of Hurricane Harvey
Source: Nat Commun. 2022 Aug 25;13:3418. doi: 10.1038/s41467-022-31056-2 (PMC9411551; doi:10.1038/s41467-022-31056-2)
Supplement: Supplementary file 2 — Reporting Summary [file 41467_2022_31056_MOESM2_ESM.pdf]

nature portfolio

Corresponding author(s): Kevin T. Smiley

Last updated by author(s): Jun 7, 2022

Reporting Summary

Nature Portfolio wishes to improve the reproducibility of the work that we publish. This form provides structure for consistency and transparency in reporting. For further information on Nature Portfolio policies, see our Editorial Policies and the Editorial Policy Checklist.

Statistics

For all statistical analyses, confirm that the following items are present in the figure legend, table legend, main text, or Methods section.

n/a	Confirmed	
☐	☒	The exact sample size (*n*) for each experimental group/condition, given as a discrete number and unit of measurement
☐	☒	A statement on whether measurements were taken from distinct samples or whether the same sample was measured repeatedly
☐	☒	The statistical test(s) used AND whether they are one- or two-sided<br>*Only common tests should be described solely by name; describe more complex techniques in the Methods section.*
☐	☒	A description of all covariates tested
☐	☒	A description of any assumptions or corrections, such as tests of normality and adjustment for multiple comparisons
☐	☒	A full description of the statistical parameters including central tendency (e.g. means) or other basic estimates (e.g. regression coefficient) AND variation (e.g. standard deviation) or associated estimates of uncertainty (e.g. confidence intervals)
☐	☒	For null hypothesis testing, the test statistic (e.g. *F*, *t*, *r*) with confidence intervals, effect sizes, degrees of freedom and *P* value noted<br>*Give P values as exact values whenever suitable.*
☒	☐	For Bayesian analysis, information on the choice of priors and Markov chain Monte Carlo settings
☐	☒	For hierarchical and complex designs, identification of the appropriate level for tests and full reporting of outcomes
☒	☐	Estimates of effect sizes (e.g. Cohen's *d*, Pearson's *r*), indicating how they were calculated

Our web collection on statistics for biologists contains articles on many of the points above.

Software and code

Policy information about availability of computer code

Data collection	Software used in data collection includes ArcGISPro 2.9.2., QGIS 3.16, and Stata 16.1
Data analysis	Software used in data collection includes ArcGISPro 2.92., QGIS 3.16, and Stata 16.1

For manuscripts utilizing custom algorithms or software that are central to the research but not yet described in published literature, software must be made available to editors and reviewers. We strongly encourage code deposition in a community repository (e.g. GitHub). See the Nature Portfolio guidelines for submitting code & software for further information.

Data

Policy information about availability of data

All manuscripts must include a data availability statement. This statement should provide the following information, where applicable:

- Accession codes, unique identifiers, or web links for publicly available datasets
- A description of any restrictions on data availability
- For clinical datasets or third party data, please ensure that the statement adheres to our policy

The data that support the findings of this study on flood area and volume are available at: https://portal.nersc.gov/cascade/Harvey/. Data on flood depths and damages at the building-level are available from Fathom but restrictions apply to the availability of these data, which were used under license for the current study, and so are not publicly available. The data are available for non-commercial academic research upon reasonable request from Fathom. Data on parcels are available from the Harris County Appraisal District: https://hcad.org/. Data on neighborhood socio-demographics are available from the National Historical Geographic Information Systems: https://www.nhgis.org/. Data on FEMA floodplain location are available from the Kinder Institute for Urban Research at Rice University: https://www.kinderudp.org/#/datasetCatalog/5je3glm092ky.

Field-specific reporting

Please select the one below that is the best fit for your research. If you are not sure, read the appropriate sections before making your selection.

- [ ] Life sciences
- [x] Behavioural & social sciences
- [ ] Ecological, evolutionary & environmental sciences

For a reference copy of the document with all sections, see nature.com/documents/nr-reporting-summary-flat.pdf

Behavioural & social sciences study design

All studies must disclose on these points even when the disclosure is negative.

Study description

This is a quantitative, cross-sectional spatial study.

Research sample

The research sample are all residential parcels in Harris County, Texas and all census tracts in Harris County, Texas. The sample is a census of all parcels and census tracts in the county and therefore representative. The rationale for the study sample is that it was a heavily affected county during Hurricane Harvey.

To detail the datasets and sources, we reproduce below the Data section in the Methods of the manuscript:
The empirical analysis we undertake in this study is based on combining information from geo-spatial data from five different sources. Geo-spatial data was analyzed in ArcGISPro 2.9.2 and QGIS 3.16.

First, data on climate change-attributed flooding comes from Wehner and Sampson's (2021) climate change-attribution hydrodynamic models [16]. To determine the effect of climate change on this baseline flood, seven scenarios of the percentage increase in precipitation based on peer reviewed research were used to calculate the spatial extent of flooding: 7% (the lowest precipitation change attribution level as set by the Clausius-Clapeyron scaling as noted by Risser and Wehner 2017 [10]); 8% (the lower bound of Van Oldenbough et al. 2017 [12]); 13% (the lower bound of Wang et al. 2018 [13]); 19% (the likely lower bound of the small region of Risser and Wehner 2017 [10] and the upper bound of Van Oldenborgh et al. 2017 [12]); 20% (best estimate by Wang et al. 2018 [13]); 24% (best estimate of the large region by Risser and Wehner 2017 [10]), and 38% (the best estimate of the small region by Risser and Wehner 2017 [10] and near the upper bound of the estimate by Wang et al. 2018 [13]).

In short, these flood maps estimate the counterfactual flood impacts from Hurricane Harvey if the identified climate change-attributed rainfall (as percent of total rainfall) did not occur. Each of these climate change attribution studies used different modeling techniques to model how climate change increased precipitation but all align in tracing how the storm's rainfall differed from historical averages due to biophysical changes; especially the increased moisture content of air and increased temperature of the Gulf of Mexico at the time of the storm. The full hydrological model used to construct the data built on these scenarios we utilize here is described in Wing et al. 2020 (20, 45).

Second, building data is sourced from the National Structure Inventory (NSI) of the U.S. Army Corps of Engineers, and representing every structure in Harris County as a point. The NSI was constructed by combining information from many datasets – including Census data, Microsoft building footprints, CoreLogic parcels, and ESRI business layers – to produce the most accurate possible inventory for assessing natural hazard risk (45). The data includes information on occupancy type, first floor elevation (including foundation type and whether the structure was built before or after the area was mapped as part of the 100-year floodplain), and the replacement value in order to link them to depth-damage functions and assess their flood vulnerability. We only include structures flooded above a 20cm threshold; as very shallow flood depths are unlikely to cause much damage from surface water or pluvial flooding. Since it is composed from proprietary data, this dataset is not publicly available. Further details can be found in (20, 46). Our damage estimates are somewhat lower than another recent study: we estimate residential damages at $6.42 billion compared to $11.1 billion in Sebastian et al. 2021 (47). We attribute these differences primarily to our calculation of building damages using replacement value instead of market value (as the latter also includes the value of the land), as well as possible differences in the depth-damage functions we use, in the modeling of riverine networks, and in the study's spatial domain. Further, we believe our use of replacement value instead of market value as a depth-damage function is justified for a few reasons. First, replacement value directly estimates impacts to residential structures and contents from flooding. This differs from approaches using market value which includes the land value that has characteristics (such as neighborhood desirability or how the land-use of the building affects the value of the land) that are not easily measured. While economic models exist that could describe damage as a function of market value such as hedonic property value modeling for cost benefit analyses relating to environmental impacts (48), our use of depth-damage functions is favored because of the closer focus on replacement value of estimated damages. Second, there are large disparities in market value based on the racial composition of neighborhoods (21, 22) meaning that a depth-damage function for market value could undercount damages in minority and/or low-income neighborhoods. Still, future research might consider other approaches such as hedonic models that take into account market value because the present approach with replacement value does not measure the land value of the parcel or how the land value of the parcel may be affected by damages to the buildings in the parcel.

Third, parcel-level data is obtained from the 2016 Harris County Appraisal District (HCAD) database. Harris County is the central county of the Houston metropolitan area. These data include more than 1.4 million parcels and are updated annually. Our study focuses only on the 1.1 million parcels which include residential property. We do not focus on commercial buildings as they are more variable in their structural vulnerabilities and their financial value, making the modeling of damage considerably more difficult. Building data is merged with the parcel data using a spatial join in GIS software. Among residential parcels that had flooded buildings, 7.7% (8,822 parcels) had multiple buildings flooded. In these cases, the depths of flooded buildings were averaged, and the damages were summed for the whole parcel.

Fourth, census-tract level data is from the five-year pooled estimates from the 2012-2016 American Community Survey (ACS). The pooled five-year estimates are used to improve the reliability of the survey. Census tracts are units commonly used in socio-economic geo-spatial research to denote neighborhoods and have approximately 4,000 residents. We obtain social and demographic data from the ACS on 798 census tracts. Three census tracts had missing values on median income, but these three tracts have only 11 parcels between them; these parcels are dropped from the study sample.

Fifth, data on FEMA-delineated floodplains is obtained for the 100-year floodplain from 2017. This area signifies places that would

	experience flood inundation in a flood event that has a 1% chance of occurring in a given year. Data was obtained from the Urban Data Platform of Rice University's Kinder Institute for Urban Research (49).
Sampling strategy	The sample comprises a census, that is all parcels and census tracts in Harris County (that met a limited set of criteria). No sample size calculation was conducted because it is a census.
Data collection	Data collection on parcels and census tracts was not conducted by the research team. The data collection for parcel data was carried out by housing appraisers as part of their routine duties. The data collection for census tracts was carried by U.S. Census officials through the American Community Survey.
Timing	The parcel data from the Harris County Appraisal District is from 2016. The census tract data from the American Community Survey is from 2012 to 2016.
Data exclusions	Data was excluded if the parcel did not have a value for the year it was built (n=7) or its appraised value (n=27). Parcels were also excluded from the analysis if they were located in one of three census tracts that did not have a value for median income (n=11).
Non-participation	Non-participation was not applicable to this study as social data is from publicly available appraisal data (i.e. the parcel data) and the census tract data is aggregated based on individual responses to the American Community Survey.
Randomization	Randomization was not appropriate for this study. We used a regression-based approach that utilized important covariates as independent variables including control variables at both the tract and parcel level.

Reporting for specific materials, systems and methods

We require information from authors about some types of materials, experimental systems and methods used in many studies. Here, indicate whether each material, system or method listed is relevant to your study. If you are not sure if a list item applies to your research, read the appropriate section before selecting a response.

Materials & experimental systems

n/a	Involved in the study
☒	☐ Antibodies
☒	☐ Eukaryotic cell lines
☒	☐ Palaeontology and archaeology
☒	☐ Animals and other organisms
☒	☐ Human research participants
☒	☐ Clinical data
☒	☐ Dual use research of concern

Methods

n/a	Involved in the study
☒	☐ ChIP-seq
☒	☐ Flow cytometry
☒	☐ MRI-based neuroimaging
